# Global prevalence of hypertensive disorders of pregnancy: a scoping review and global perspective by country and region

**DOI:** 10.3389/fcvm.2026.1692590

**Published:** 2026-04-22

**Authors:** Yunjie Li, Rana F. Chehab, Emily Z. Wang, Sita Manasa Susarla, Clara Voong, Hongyi Chen, Mariana C. Cabatu, Hadley Sorsby-Jones, Yeyi Zhu

**Affiliations:** 1 Kaiser Permanente Bernard J. Tyson School of Medicine, Pasadena, CA, United States; 2 Division of Research, Kaiser Permanente Northern California, Pleasanton, CA, United States; 3 Kaiser Permanente Northern California, Center for Upstream Prevention of Adiposity and Diabetes Mellitus (UPSTREAM), Pleasanton, CA, United States; 4 School of Public Health, University of California, Berkeley, Berkeley, CA, United States; 5 School of Public Health, Columbia University, New York, NY, United States; 6 Department of Epidemiology & Biostatistics, University of California, San Francisco, CA, United States

**Keywords:** eclampsia, global prevalence, hypertension, hypertensive disorders of pregnancy, preeclampsia, pregnancy

## Abstract

**Introduction:**

Hypertensive disorders of pregnancy (HDP) are among the leading causes of maternal morbidity and mortality worldwide and an increasing global concern. This review aimed to estimate the global prevalence of HDP by country and World Health Organization (WHO) region using nationally representative, population-based studies.

**Methods:**

We searched PubMed and Embase for articles published between January 2004 and December 2023 and included population-based, observational studies with nationally or internationally representative samples reporting primary data on HDP prevalence. Study quality was assessed using a modified version of the Newcastle-Ottawa Scale.

**Results:**

A total of 143 studies from 31 countries across six WHO regions met the inclusion criteria. The African Region reported the highest prevalence of chronic hypertension (4.80%), gestational hypertension (17.50%), and composite HDP (22.20%), although nationally representative prevalence estimates for preeclampsia and eclampsia were not available for this region. In contrast, the Eastern Mediterranean Region reported the lowest rates of chronic hypertension (1.10%), gestational hypertension (1.70%), preeclampsia (1.45%), and HDP (5.10%). The European Region had intermediate rates of chronic hypertension (1.70%), gestational hypertension (2.90%), preeclampsia (2.50%), eclampsia (0.02%) and HDP (5.90%). The Americas Region and the Western Pacific Region had varying rates across the HDP spectrum: chronic hypertension (2.35%, 3.15%), gestational hypertension (5.10%, 1.98%), preeclampsia (5.35%, 2.45%), eclampsia (0.08%, 0.06%), and HDP (8.04%, 5.60%, respectively). High-income countries had more studies of higher methodological quality than low- and middle-income countries.

**Discussion:**

We observed considerable variability in HDP prevalence and study quality across countries and regions. This may stem from variations in screening methods, diagnostic criteria, and data availability, highlighting critical gaps in standardized protocols particularly in resource-limited settings.

## 1 Introduction

Hypertensive disorders of pregnancy (HDP) are the leading causes of maternal morbidity and mortality worldwide (1). HDP represents a spectrum of conditions characterized by elevated blood pressure during pregnancy, including chronic hypertension, gestational hypertension, preeclampsia, and eclampsia (2). Understanding the global burden of HDP is of substantial public health importance for identifying at-risk populations, prioritizing research, and informing public health strategies to improve maternal and child health outcomes.

Recent data suggest a concerning rise in the incidence of HDP. Among singleton deliveries in the United States between 1989 and 2020, the incidence of HDP significantly increased by 3.6% annually (95% confidence interval: 3.0% to 4.1%) (3). Similarly, an age-period-cohort analysis based on the Global Burden of Disease (GBD) Study reported a 15.2% increase in the global HDP incidence and a 487.0% increase in prevalence between 1990 and 2021 (4, 5). The rising trend in HDP may be attributed to factors including the obesity epidemic and increasing maternal age, which will likely continue, contributing to an emerging worldwide epidemic (6).

Therefore, estimating the prevalence of HDP on a global scale is critical for developing and implementing prevention policies and improving maternal and fetal outcomes. Although previous efforts based on the GBD Study have sought to characterize the global burden of HDP (4, 5, 7, 8), these studies may not fully capture the true prevalence due to limitations in data sources and methodology, including but not limited to indirect prevalence inference from available mortality data or related indicators rather than direct estimates from population-based studies. More importantly, previous studies focused on the prevalence of HDP as a composite disease; however, HDP comprise a wide spectrum of severity and phenotypes (9). Estimating the global prevalence of individual HDP conditions is crucial for better understanding their distinct disease burdens and guiding tailored prevention and management strategies.

In this review, we aimed to provide a comprehensive overview of data published over the last two decades to estimate the global burden of the entire HDP spectrum, focusing on population-based, nationally representative studies that used direct prevalence assessment rather than statistical estimations. In addition, we highlighted the methodological challenges involved in estimating its global burden.

## 2 Materials and methods

### 2.1 Search strategy

We conducted a comprehensive literature search across PubMed and Embase to identify relevant studies published between January 1, 2004, and December 31, 2023. We used keywords “hypertension, pregnancy”, “preeclampsia/pre-eclampsia”, “eclampsia”, “prevalence”, or “incidence” as MeSH terms or text words, “hypertension, pregnancy-induced” as MeSH terms, “high blood pressure”, “hypertensive”, “gestation”, “gestational hypertension”, or “gravidity” as text words in the final search (see the full list in Appendix 1). We followed the PRISMA guidelines and registered the protocol (CRD42023437298) on PROSPERO (http://www.crd.york.ac.uk/prospero) (10).

### 2.2 Screening and eligibility

**Figure 1:**
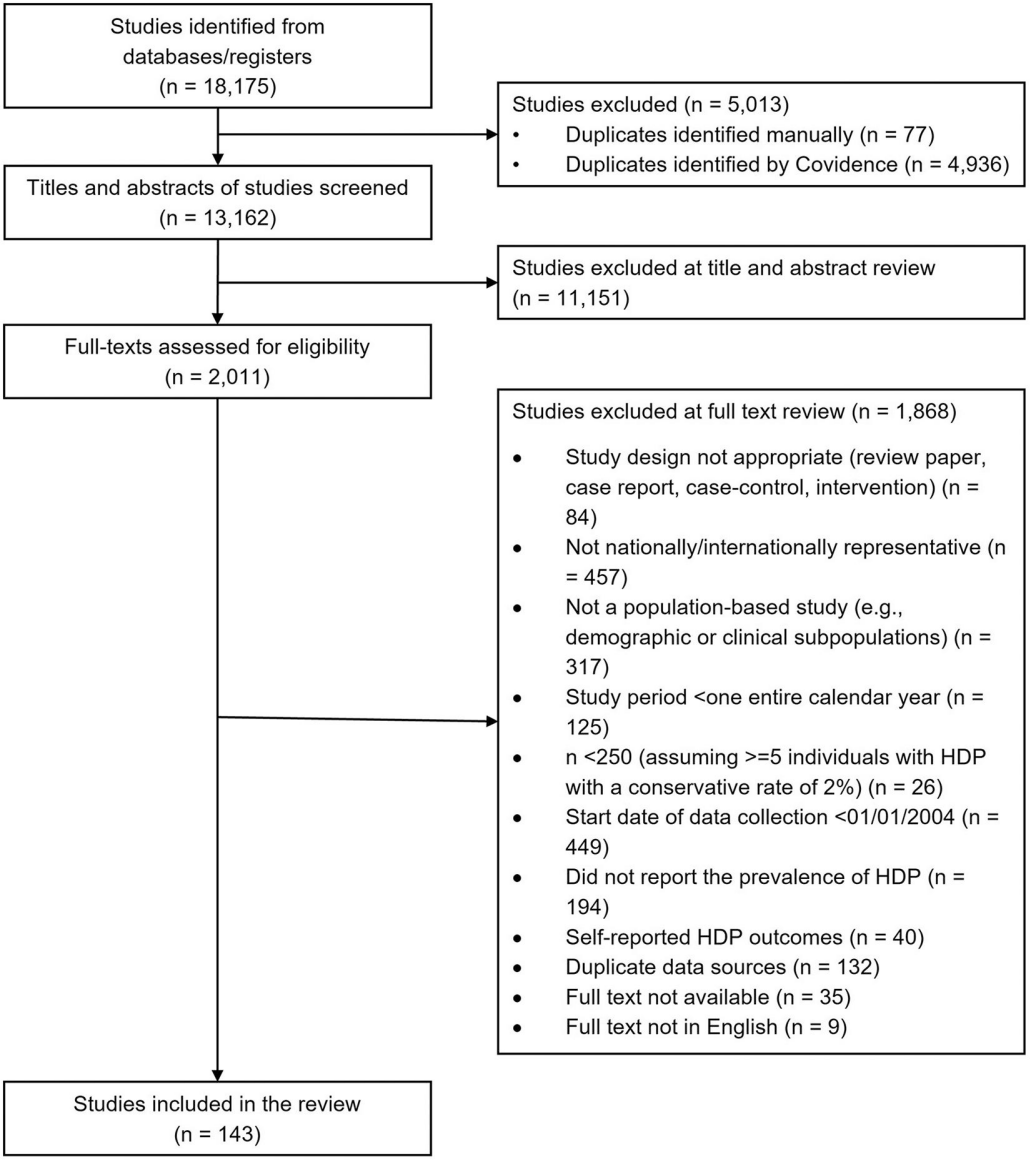
Study flowchart. HDP, hypertensive disorders of pregnancy.

A total of 18,175 records were identified through database searches utilizing the above search strategy (Figure 1). Eligible studies included population-based observational (prospective or retrospective cohort or cross-sectional) studies with study sample representative of national or international populations reporting primary data on the prevalence of HDP (i.e., chronic hypertension, gestational hypertension, preeclampsia, eclampsia, or any combination of these disorders). We conducted article screening using the Covidence software. A total of 5,013 duplicates were removed before the screening process, yielding 13,162 studies for title and abstract screening for potential relevance. The screening was completed by two separate reviewers, with conflicts resolved by a third reviewer. During the title and abstract screening phase, we excluded 11,151 studies that did not meet our inclusion criteria listed above and exclusion criteria listed below, with 2,011 articles remaining for the full-text review assessed for eligibility. During the full text screening phase, we excluded studies that were reviews, case reports, case-control studies, or interventional trials (*n* = 84); were not nationally representative (*n* = 457) or population-based (*n* = 317); had a study period of less than one calendar year (*n* = 125); had a sample size below 250 (*n* = 26); began data collection before January 1, 2004 (*n* = 449); did not report HDP prevalence (*n* = 194); relied on self-reported HDP outcomes (*n* = 40); had duplicate data sources (*n* = 133); did not have full-text (*n* = 35), or involved non-English data (*n* = 9). In total, 143 eligible studies met the eligibility criteria; data were reviewed and extracted data from these studies (see the full list of studies in Appendix 2).

### 2.3 Data extraction and synthesis

Data on study aim(s), study country/jurisdictions, study period, study design, definition of HDP, sample size, and HDP prevalence rates were extracted. Two independent reviewers extracted data in duplicate using Covidence's Extraction 2.0 tool, and any discrepancies were resolved through adjunction by a third reviewer. Prevalence estimates were synthesized by country/jurisdictions and the World Health Organization (WHO) regions, respectively, and stratified by specific HDP subtypes where possible. For countries/jurisdictions with multiple studies during the study period of interest, the median prevalence value was calculated to represent the country/jurisdiction-specific HDP prevalence from 2004 to 2023. Further, the median of these country/jurisdiction-specific prevalence estimates was used to calculate the WHO region-specific HDP prevalence across 2004–2023.

### 2.4 Quality assessment

We used a quality assessment scale adapted from the Newcastle-Ottawa Scale (11). Specifically, the quality of the data source was scored according to sampling strategy (random sampling, systematic sampling, stratified sampling, or cluster sampling vs. not specified; scored 1 vs. 0), study sample size (n≥2500 vs. *n* < 2500; scored 1 vs. 0), assessment of HDP (diagnosed by health professional, diagnosed in the field, extracted from birth certificates/surveillance record vs. no description; scored 1 vs. 0), age-standardized HDP prevalence (yes vs. no; scored 1 vs. 0), and report of HDP specification (only one HDP type or individual HDP subtypes vs. composite HDP including ≥2 subtypes; scored 1 vs. 0). Scores of the 5 categories were aggregated, and a final quality score was assigned to each study (poo*r* = 0–1, fai*r* = 2–3, good=4–5) (Supplementary Table 1).

## 3 Results

### 3.1 HDP definitions

We provided a synthesis of commonly used definitions from leading clinical guidelines (Table 1), as well as the specific definitions employed by individual studies when available (Supplementary Table 2). Most clinical practices follow guidelines from the American College of Obstetricians and Gynecologists (9, 12), the International Society for the Study of Hypertension in Pregnancy (13), and other national bodies including National Institute for Health and Care Excellence (14), Society of Obstetricians and Gynecologists of Canada (15), and Society of Obstetric Medicine of Australia and New Zealand (16). Although definitions of HDP subtypes may vary internationally, the commonly accepted categories include chronic hypertension before pregnancy or during early pregnancy (<20 weeks of gestation), gestational hypertension (≥20 weeks of gestation), preeclampsia/eclampsia (≥20 weeks of gestation), and chronic hypertension with superimposed preeclampsia (16). Some guidelines also recognize white coat hypertension (13, 16–18), while others classify eclampsia and hemolysis, elevated liver enzymes, and low platelet count syndrome as a distinct subtype separate from preeclampsia (14, 19).

**Table 1:** Definition of hypertensive disorders of pregnancy across international guidelines

Disorder	American College of Obstetricians and Gynecologists (United States) (9, 12)	International Society for the Study of Hypertension in Pregnancy (13)	National Institute for Health and Care Excellence (United Kingdom) (14)	Society of Obstetricians and Gynecologists of Canada (Canada) (15)	Society of Obstetric Medicine of Australia and New Zealand (Australia, New Zealand) (16)
Chronic hypertension	Hypertension diagnosed before pregnancy, before 20 weeks gestation, or during pregnancy that does not resolve in the postpartum period (3 months after childbirth)	Hypertension diagnosed before 20 gestational weeks, with 2 measurements at least 4 hours apart during the same visit or in two consecutive visits	Hypertension that is present at the booking visit, or before 20 weeks, or if the woman is already taking antihypertensive medication when referred to maternity services. It can be primary or secondary in etiology.	Hypertension that develops either before pregnancy or at <20 weeks.	Hypertension confirmed either before pregnancy or before 20 completed weeks gestation. The chronic hypertension may be either: Primary (or essential) hypertension, or secondary hypertension.
Gestational hypertension	Hypertension diagnosed after 20 weeks gestation, without elevated blood pressure readings prior, and without proteinuria or any other features listed under preeclampsia	New-onset hypertension diagnosed at or after 20 gestational weeks in the absence of features of preeclampsia	New hypertension presenting after 20 weeks of pregnancy without significant proteinuria.	Hypertension that develops for the first time at ≥20 weeks without evidence of preeclampsia.	New onset of hypertension after 20 weeks with no evidence of end organ involvement.
Preeclampsia^a^	Gestational hypertension and any of the following coexisting feature (urinary, laboratory, or symptom): Urinary finding: ● ≥ 300 mg urine protein/24 hours ● Protein to creatinine ratio ≥ 0.3 ● Urine dipstick of 2+ if the first two values cannot be obtained Laboratory finding: ● Thrombocytopenia (platelet < 100 × 10^9^/L) ● Renal insufficiency (serum creatinine > 1.1 mg/dL or 2× of baseline in the absence of renal disease) ● Impaired liver function (2× of baseline or upper limit of normal aspartate aminotransferase or alanine aminotransferase) Symptom (new onset, unresponsive to medication, cannot be attributed to any other condition): ● Right upper quadrant or epigastric pain ● Pulmonary edema ● Headache ● Visual disturbances	Gestational hypertension with at least one of the following: ● Proteinuria ● Other maternal organ dysfunctions, including: ○ Acute kidney injury ○ Liver involvement (alanine aminotransferase or aspartate aminotransferase > 40 IU/L) with or without right upper quadrant or epigastric abdominal pain ○ Neurological complications (eclampsia, altered mental status, blindness, stroke, clonus, severe headache, persistent visual scotomata) ○ Hematological complications (thrombocytopenia, disseminated intravascular coagulation, hemolysis) ● Uteroplacental dysfunction (intrauterine growth restriction, abnormal umbilical artery Doppler waveform analysis, or stillbirth)	New onset of hypertension (over 140 mmHg systolic or over 90 mmHg diastolic) after 20 weeks of pregnancy and the coexistence of 1 or more of the following new-onset conditions: ● Proteinuria (urine protein: creatinine ratio of 30 mg/mmol or more or albumin: creatinine ratio of 8 mg/mmol or more, or at least 1 g/liter (2+) on dipstick testing) ● Other maternal organ dysfunction ○ Renal insufficiency ○ Liver involvement ○ Neurological complications ○ Hematological complications Uteroplacental dysfunction such as fetal growth restriction, abnormal umbilical artery doppler waveform analysis, or stillbirth	Gestational hypertension with new-onset proteinuria or one/more adverse conditions (defined as a maternal end-organ complication or evidence of uteroplacental dysfunction).	Multisystem disorder. New onset hypertension (≥140 and/or ≥90mmHg) after 20 weeks gestation accompanied by one or more of the following new onset organ involvements: ● Renal Involvement ○ Proteinuria (spot urine/protein: creatinine ratio ≥30 mg/mmol) ○ Serum creatinine ≥90µmol/L) ● Liver Involvement ○ Raised serum transaminases (in the absence of alternative diagnosis) ● Hematological Development ○ Thrombocytopenia (<150,000µ/L) ○ Feature of hemolysis: decreased haptoglobin with or without fragmented red cells, elevated lactate dehydrogenase) ○ Disseminated intravascular coagulation ● Neurological Development ○ Seizure ○ Features of cerebral irritability ○ Cerebrovascular Accident ● Pulmonary Edema Features of Placental Dysfunction
Chronic hypertension superimposed with preeclampsia	Preeclampsia in pregnant individual with a history of chronic hypertension	Any of the maternal organ dysfunctions consistent with preeclampsia developing in chronic hypertensive patients or new-onset proteinuria accompanied by a rise in blood pressure		Development of 1 or more characteristics of preeclampsia (i.e., new-onset proteinuria or 1 or more adverse complications) superimposed on chronic hypertension.	Features of preeclampsia superimposed on either pre-existing hypertension or renal disease.
Eclampsia	New onset seizure(s) (tonic-clonic, focal, multifocal) that cannot be attributed to any other condition (epilepsy, cerebral arterial ischemia and infarction, intracranial hemorrhage, or drug use)		A convulsive condition associated with preeclampsia.		

a Preeclampsia by American College of Obstetricians and Gynecologists definition further includes subtypes of 1) severe feature and 2) hemolysis, elevated liver enzymes, and low platelet count syndrome.

Similarly, although diagnostic criteria may vary between guidelines, they generally converge on defining hypertension as a systolic blood pressure ≥140 mmHg and/or diastolic blood pressure ≥90 mmHg, while severe hypertension is defined as systolic blood pressure ≥160 mmHg and/or diastolic blood pressure ≥110 mmHg. In most cases, two elevated blood pressure readings at different occasions are required to confirm the diagnosis.

Chronic hypertension is defined as hypertension diagnosed before 20 weeks of gestation, whereas gestational hypertension, preeclampsia, and eclampsia are diagnosed afterward. Preeclampsia is characterized by hypertension accompanied by signs of maternal end-organ damage or uteroplacental dysfunction, while eclampsia is the convulsive manifestation of preeclampsia (9, 12). Notably, HDP is often described as a spectrum, where conditions may evolve. For instance, a patient with chronic hypertension may develop proteinuria or end-organ damage after 20 weeks of gestation, leading to a diagnosis of chronic hypertension with superimposed preeclampsia. This progression can significantly impact management strategies, including the potential need for early delivery with induction of labor (16).

### 3.2 National and within-region prevalence of HDP

We summarized the prevalence of the HDP spectrum from 143 studies (Supplementary Table 3) across 31 countries and stratified by WHO regions (Supplementary Table 4). It is important to note that the studies varied in the subtype(s) of HDP reported, including chronic hypertension, gestational hypertension, preeclampsia, eclampsia, combined preeclampsia/eclampsia, combined gestational hypertension/preeclampsia/eclampsia, all types of HDP (chronic hypertension, gestational hypertension, preeclampsia, eclampsia), and other categories specified by some studies.

**Figure 2:**
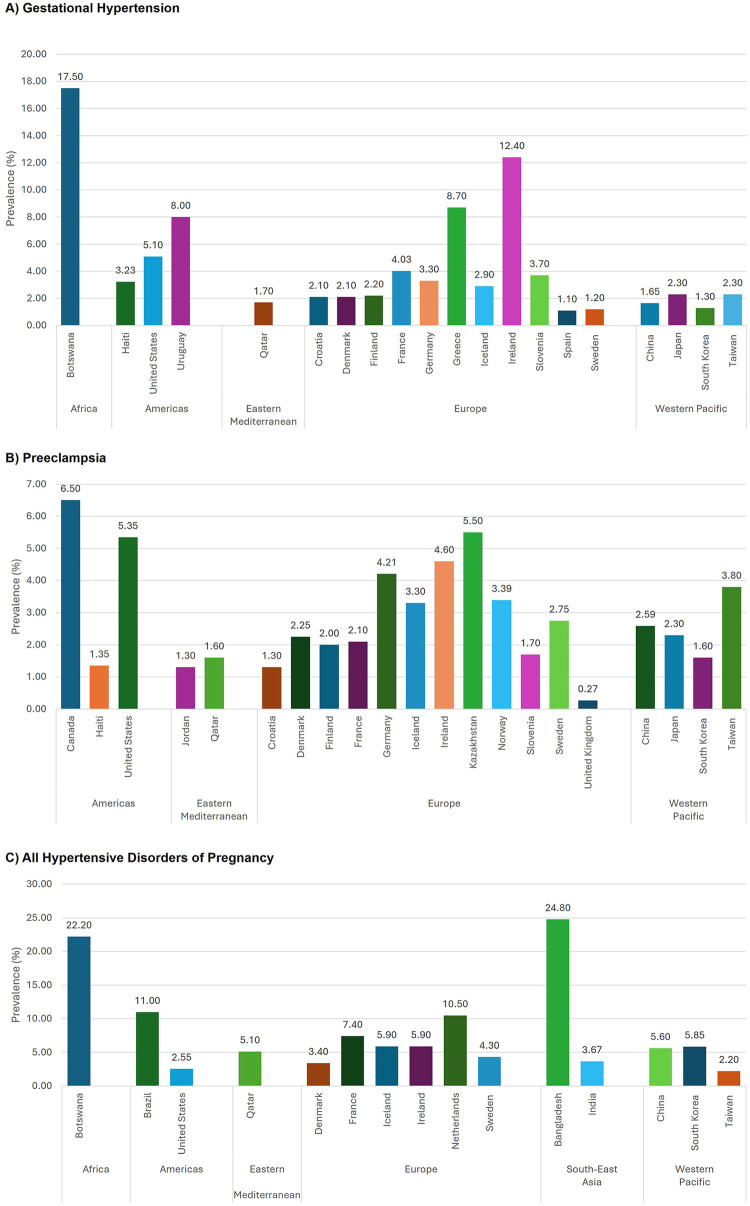
Global prevalence of **(A)** gestational hypertension, **(B)** preeclampsia, **(C)** all hypertensive disorders of pregnancy by country/jurisdictions.

There were notable variations in gestational hypertension, preeclampsia, and overall HDP prevalence across countries (Figure 2, Supplementary Table 4). For gestational hypertension, Botswana reported the highest prevalence (17.50%), while Spain had the lowest prevalence (1.10%) (Figure 2A). There were multiple eligible, country-specific studies reported in the Americas, Europe, and Western Pacific Regions. In the Americas Region, prevalence estimates of gestational hypertension ranged from 3.23% in Haiti to 8.00% in Uruguay. Similarly, within the European Region, Ireland reported the highest prevalence of gestational hypertension (12.40%), approximately twelve times that of Spain (1.10%). In contrast, the prevalence estimates for the four countries/jurisdictions (China, Japan, South Korea, and Taiwan) in the Western Pacific Region showed little variation, ranging from 1.30% to 2.30%.

For preeclampsia, the highest prevalence was reported in Canada (6.50%), while the United Kingdom had the lowest (0.27%) (Figure 2B). In the Americas Region, the United States (5.35%) had a rate similar to that of Canada, whereas Haiti had a lower rate (1.35%). In the European Region, Ireland again showed the highest prevalence of preeclampsia (4.60%), almost seventeen times that of the United Kingdom (0.27%). Preeclampsia rates were more consistent within the Eastern Mediterranean (1.30–1.60%) and the Western Pacific (1.30–2.30%) Regions.

For overall HDP (chronic hypertension, gestational hypertension, preeclampsia, eclampsia), Bangladesh reported the highest prevalence (24.80%), followed closely by Botswana (22.20%) (Figure 2C). Taiwan had the lowest rate of all HDP (2.20%). Within some regions, there was wide variation across individual countries in the prevalence of individual and composite HDP conditions. For example, in the Americas Region, the United States reported an all HDP prevalence of 2.55% contrasted with Brazil's much higher prevalence of 11.00%. Within the European Region, Netherlands had the highest prevalence of all HDP (10.50%) compared to the lowest in Denmark (3.40%). In the South-East Asian Region, the prevalence in Bangladesh (24.80%) was more than six times that of India (3.67%). In the Western Pacific Region, estimates were more consistent, ranging from 2.20% in Taiwan to 5.85% in South Korea. Similarly, significant variability in other HDP conditions (chronic hypertension, eclampsia, and various combinations of individual HDP conditions) were observed across countries (Supplementary Figure 1).

### 3.3 Across-region prevalence of HDP

**Figure 3:**
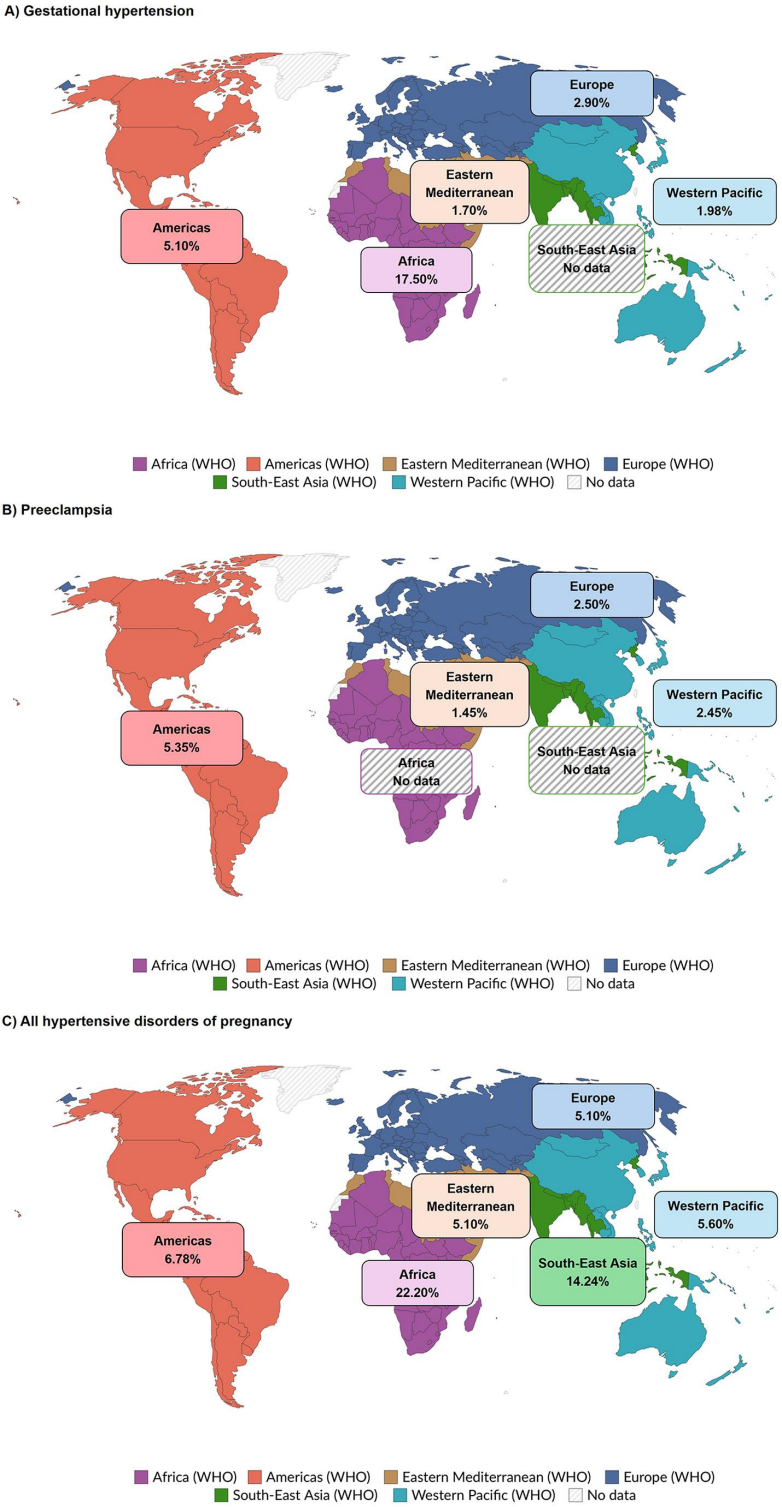
Global prevalence of **(A)** gestational hypertension, **(B)** preeclampsia, and **(C)** all hypertensive disorders of pregnancy by WHO region.

Similar to the variability within WHO regions, differences in the prevalence of HDP were observed across WHO regions (Figure 3, Supplementary Table 5, Figure 2). The African Region reported the highest prevalence of gestational hypertension at 17.50%, followed by the Americas Region (5.10%) and the European Region (2.90%). The Eastern Mediterranean and the Western Pacific Regions had similarly low rates at 1.70% and 1.98%, respectively (Figure 3A). For preeclampsia, the highest prevalence was observed in the Americas Region (5.35%), followed by the European (2.50%), the Western Pacific (2.45%), and the Eastern Mediterranean (1.45%) Regions (Figure 3B). The African Region exhibited the highest overall burden of HDP at 22.20%, followed by the South-East Asian Region (14.24%). In contrast, the Americas Region (6.78%), the European (5.90%), the Western Pacific (5.60%), and the Eastern Mediterranean (5.10%) Regions reported lower overall HDP prevalences (Figure 3C). Significant variability in other HDP conditions (chronic hypertension, eclampsia, and various combinations of individual HDP conditions) were observed across WHO regions (Supplementary Figure 2).

### 3.4 Research quality by country and region

Study quality varied across countries and WHO regions (Table 2), with detailed quality assessment breakdowns for all studies available in Supplementary Table 6. The African Region had only one included study (good quality), from Botswana, which is an upper-middle income country. Most countries in the African Region are low- or lower-middle income, which may contribute to underrepresentation of high-quality, large-scale studies due to limited research infrastructure and resources (Supplementary Table 7). The Eastern Mediterranean Region was represented by three studies, two from Qatar (good and fair quality), a high-income country, and one from Jordan (fair quality), a lower-middle income country. The South-East Asia Region composed predominantly of lower-middle income countries, was represented by four studies: one from Bangladesh (poor quality) and three from India (100% fair quality) (20).

**Table 2:** Summary of quality of studies by country/jurisdictions and World Health Organization region.

WHO region and country/jurisdictions	Study quality			Total, *N*
	Good (%)	Fair (%)	Poor (%)	
African Region				**1**
Botswana	100			1
Americas Region				**57**
Brazil	50	50		2
Canada	67	33		3
Haiti	100			1
Suriname		100		1
United States	67	33		49
Uruguay	100			1
Eastern Mediterranean Region				**3**
Jordan		100		1
Qatar	50	50		2
European Region				**42**
Croatia	100			1
Denmark		100		2
Finland	50	50		4
France	50	50		10
Germany	33	33	33	3
Greece		100		3
Iceland	100			1
Ireland	50	50		2
Kazakhstan		100		1
Netherlands		100		2
Norway		100		1
Slovenia	100			1
Spain		100		1
Sweden	25	75		8
United Kingdom		50	50	2
South-East Asia Region				**4**
Bangladesh			100	1
India		100		3
Western Pacific Region				**36**
Australia		100		1
China	40	40	20	5
Japan	50	50		2
South Korea	26	74		19
Taiwan	56	44		9

Numbers representing the sum of studies per WHO region were bolded.

In contrast, the Americas Region had the highest number of studies (*n* = 57), driven primarily by the United States (*n* = 49), where 67% were rated as good quality and 33% as fair. The majority of studies in the Americas Region (*n* = 56) originated from upper-middle and high-income countries (20), with Haiti being the only lower-middle income country represented; its single study was rated as good quality.

The European Region contributed with 42 studies, which were distributed more evenly across 15 countries. France (*n* = 10) and Sweden (*n* = 8) had higher numbers of studies. Several countries (Croatia, Iceland, Slovenia) had 100% good-quality studies, though each of these had only one study included. Most other European countries showed variability in quality, typically between good and fair. Nearly all countries in the European Region were high-income, with the exception of Kazakhstan, an upper-middle income country (20).

Lastly, in the Western Pacific Region (*n* = 36), South Korea contributed the largest number of studies (*n* = 19), with 26% rated as good and 74% as fair quality. Other countries in the Western Pacific Region also demonstrated substantial heterogeneity in study quality. While the region as a whole includes a range of income levels, all countries represented in the analysis were high-income with the exception of China, classified as upper-middle income (20).

## 4 Discussion

### 4.1 Summary of findings

This review highlights substantial variations in the prevalence of HDP globally, the diverse patterns of which may reflect differences in type(s) of HDP reported, screening approaches, diagnostic criteria, and study populations. The highest national prevalence rates were observed in Botswana for gestational hypertension (17.5%), Germany for chronic hypertension (6.1%), Canada for preeclampsia (6.5%), Suriname for eclampsia (0.78%), and Bangladesh for all HDP (24.8%). Regionally, the African Region had the highest all HDP burden (22.2%), while the Eastern Mediterranean Region had the lowest (5.1%). Study quality also varied widely, with the majority of the data derived from upper-middle and high-income countries; low-income countries remained underrepresented. Our findings reveal the need for standardized diagnostic criteria for HDP as well as increase in research resources in low-resource settings to ensure accurate estimation of the global burden and inform targeted prevention strategies. In countries with robust hospital or insurance networks, we believe that development of comprehensive databases may better capture HDP prevalence and facilitate disease surveillance and monitoring within these systems.

### 4.2 Comparison with previous studies

Synthesized data on the global prevalence of HDP, both as individual conditions and as a composite, remain limited, compounded by a lack of summary reporting and wide variation in screening and diagnostic criteria. Although the burden of HDP has been documented in studies based on the GBD Study (4, 5, 7, 8), some inherent limitations warrant further examination of the global disease burden of HDP. In countries with limited healthcare infrastructure, the GBD study often infers disease prevalence indirectly from available mortality data or related indicators, rather than utilizing direct prevalence data from population-based studies. These inferences rely on statistical methods that analyze disease progression, risk factors, and relationships between data sources, which may not accurately reflect the true prevalence of the disease in specific populations. In addition, data quality issues such as underreporting in low-resource settings and varying diagnostic criteria further complicate global comparisons. Further, prior studies reported HDP as a composite condition, despite substantial heterogeneity across HDP subtypes. We summarized the global prevalence of individual and composite HDP conditions by country and WHO region and the corresponding diagnosis criteria (9).

### 4.3 HDP screening approaches

The variations we observed in the global prevalence of HDP may be due, in part, to differences in screening approaches. Common screenings typically involve blood pressure monitoring at prenatal visits, with urine protein testing after 20 gestation weeks. Variations exist across countries and year. For example, the United States Preventative Services Task Force broadened the scope from screening for preeclampsia in 2017 to screening for all HDP in 2023 in asymptomatic pregnant individuals throughout pregnancy (21). Similarly, the Society of Obstetric Medicine of Australia and New Zealand recommends screening all pregnant persons early in the antenatal period for their risk of developing preeclampsia, including monitoring blood pressure values at each antenatal visit (16). As part of the recommendation for preeclampsia screening, Society of Obstetric Medicine of Australia and New Zealand includes both high-risk and low-risk factors for the development of preeclampsia, which may be used in conjunction with first-trimester screening to stratify an individual's risk of developing preeclampsia. Of note, varying screening methods can contribute to underdiagnosis of HDP in regions utilizing limited or lenient criteria. For instance, a cross-sectional study in Thailand comparing algorithms of the National Institute for Health and Care Excellence and Fetal Medicine Foundation found differences in preeclampsia detection rates, diagnosing 171 vs. 201 cases (p = 0.018) (22).

### 4.4 HDP diagnostic criteria

The lack of uniformity in diagnostic guidelines and definitions of HDP may also contribute to the inconsistent prevalence estimates we observed across studies and regions, limiting comparability and potentially impacting diagnosis, management, and clinical outcomes. In addition, heterogeneity in disease definitions undermines the ability to synthesize evidence across studies and reduces generalizability of findings. To address this, we systemically recorded and aggregated data from studies reporting across the HDP spectrum, noting the specific definitions utilized by these studies to achieve a comprehensive overview. While this approach may present challenges in directly comparing results on a global scale due to differences in disease classification and diagnostic criteria, it provides a more nuanced and inclusive characterization of HDP burden across populations worldwide.

### 4.5 Contextual population characteristics in the broader literature

This review synthesized prevalence estimates and did not include individual-level exposure or risk factor data. Accordingly, the following considerations are provided as general context from the broader literature and should not be interpreted as explanations for geographic patterns observed in this review.

Advanced maternal age (AMA), defined as age ≥35 years, has been associated with increased risk for HDP in other studies. For instance, women ≥40 years old have more than threefold increased odds of preeclampsia compared to younger counterparts (23). The proportion of women giving birth at older ages varies widely across countries due to socioeconomic and cultural factors. Among low- and middle-income countries, Afghanistan has the highest prevalence of AMA births at 22%, whereas Nepal has the lowest rate of AMA births at 3% (24). Several countries in the African Region have both high rates of AMA births and HDP prevalence (e.g., Democratic Republic of Congo at 18%, Nigeria at 16%, Niger at 14%, and Angola at 13%) (24). Notably, some regions included in this review have higher proportions of births at older maternal ages, a factor that has been associated with HDP risk in prior literature. However, because age distributions were not consistently reported across included studies, this review could not assess whether differences in maternal age contributed to the observed geographic variation in HDP prevalence.

Economic status is another important risk factor associated with HDP prevalence. For example, one study in Pittsburgh reported that people living in the most economically disadvantaged neighborhoods were twice as likely to experience postpartum hypertension as those living in the most advantaged neighborhoods (25). Although the underlying mechanisms linking low socioeconomic status to increased prevalence of HDP remain to be elucidated, potential contributors may include lack of or limited access to healthcare (26), lower income (26), and increased stress related to living in a disadvantaged neighborhood (27). Socioeconomic conditions vary substantially across and within regions and have been associated with HDP risk in prior studies. However, the studies included in this review did not provide harmonized measures of socioeconomic status, and therefore this review could not evaluate its contribution to geographic variation in HDP prevalence. For context, the distribution of country income levels differs across WHO regions. For instance, from 2002–2012, 40% of the African Region countries were classified by the World Bank as low-income, compared to 14% of the Eastern Mediterranean Region countries (28). Regions such as the South-East Asia Region and the Americas Region, although lacking low-income countries, exhibit considerable economic diversity with higher proportions of lower-middle and upper-middle income countries (28).

Race and ethnicity – social, not biological, constructs – are associated with disparities in HDP prevalence, which may reflect the role of social and structural determinants of health (25). In the United States, Black individuals experience disproportionately high maternal morbidity and perinatal mortality compared to other racial/ethnic groups (21). Furthermore, two-thirds of Black individuals who are diagnosed with preeclampsia have severe features while fewer than half of White individuals with preeclampsia develop severe features (21). Similarly, Native American/Alaska Native individuals in the United States have significantly higher rates of pregnancy-related maternal morbidity and mortality compared to other racial and ethnic groups (21). The WHO acknowledges that Americas Region has high levels of inequality in access to healthcare across society (29). Therefore, regional aggregate of HDP prevalences in our report, such as that in the Americas Region, may be obscured. Although less information is available on racial and ethnic differences in other WHO regions, additional research on intra-national distribution of HDP could further characterize within-region heterogeneity in HDP prevalence.

Finally, other biological or clinical risk factors including obesity, nulliparity, pre-existing diseases (e.g., diabetes mellitus, thrombophilia, systemic lupus erythematosus, antiphospholipid antibody syndrome, chronic kidney disease, and obstructive sleep apnea), individual or family history of preeclampsia, individual history of gestational diabetes mellitus, and multifetal pregnancy have been associated with HDP risk (30). Accounting for such differences is essential when comparing rates and designing context-specific prevention strategies. Future studies integrating standardized prevalence estimates with individual-level or population-level exposure data are needed to evaluate potential contributing factors to geographic variation in HDP.

### 4.6 Strengths and limitations

The main strength of this paper lies in the utilization of population-based, nationally representative studies to better understand the role of HDP on a global level. Rather than relying on statistical estimations, we drew from two decades of published data, encompassing 143 studies across 31 countries, with each WHO region being represented by at least one study. This comprehensive dataset allowed for an accurate estimation of the global disease burden of HDP conditions and highlighted data paucity especially in resource-limited settings with high disease burden, which may inform future targeted efforts in HDP surveillance on a global scale. Further, rather than aggregating various HDP subtypes into a single composite condition, we recognized the substantial heterogeneity across individual HDP subtypes in terms of pathophysiology, diagnosis criteria, clinical manifestation, and disease progression. These differences warrant consideration of each HDP subtype as a distinct clinical entity. This study therefore provided a comprehensive, novel review of the global burden of individual HDP subtypes as well as the composite HDP conditions based on concrete, published data from nationally representative studies.

Our study also faces several limitations that should be considered. Although our review synthesizes nationally representative population-based studies from multiple WHO regions, the evidence base covers only 31 countries out of 195 sovereign states in the world and some regions remain sparsely represented. Therefore, our findings should be interpreted as reflecting the currently available evidence rather than a fully representative estimate of global HDP prevalence. Nonetheless, the 31 countries included in this review collectively represent approximately 4.2 billion individuals (over half of the global population of 8.2 billion), which provides meaningful insights into HDP prevalence patterns. In addition, HDP comprise a spectrum of conditions that may progress from one to another or coexist simultaneously. The studies included in this review varied in types of HDP conditions reported, complicating direct comparisons across studies, countries, and regions. In addition, the studies reviewed exhibit high heterogeneity in disease definitions and diagnostic criteria. To address this, we summarized commonly used definitions from leading clinical guidelines, as well as the specific definitions employed by individual studies when available. While standardized diagnostic protocols are crucial for improving comparability, we acknowledge that such standardization may be challenging given the variability in population characteristics and healthcare infrastructure. Nonetheless, efforts to standardize diagnostic criteria could reduce variability and facilitate more accurate pooled analyses for future studies.

Study quality was assessed using a modified version of the Newcastle-Ottawa Scale adapted for observational population-based studies. While this approach allowed us to evaluate key methodological features such as sampling strategy, outcome assessment, and sample size, we acknowledge that risk-of-bias tools specifically developed for prevalence studies (e.g., Hoy et al.) may offer additional advantages for assessing the methodological quality of prevalence estimates (31). Another limitation stems from the methodological limitations inherent in studies conducted in low- and middle-income countries. In these settings, research is often constrained by limited resources, smaller sample sizes, and variable study quality. Contributing factors may include inadequate healthcare infrastructure, restricted funding, and limited access to diagnostic tools or trained personnel. Consequently, the evidence from these settings may be less robust, reducing the generalizability of our findings. In addition, the literature search was limited to PubMed and Embase and restricted to English-language publications. Although these databases capture the majority of biomedical research, relevant studies published in other language or indexed in regional databases may have been missed, which potentially further contributed to underrepresentation of certain geographic regions. Future research should prioritize multilingual systematic searches, inclusion of regional databases, and strengthen international efforts to improve research capacity and healthcare infrastructure in low- and middle-income countries, which are essential to more accurately capture the global burden of HDP.

Lastly, our study used median values to summarize country/jurisdiction- and WHO region-level HDP rates instead of a formal meta-analysis. While medians effectively describe central tendencies and are less influences by extreme values, they may not fully reflect the variability or account for potential methodological biases across studies. However, the high level of heterogeneity in study design, data collection methods, diagnostic criteria, underlying population characteristics, and study quality, rendered a meta-analytic approach inappropriate for this review. In addition, HDP prevalence estimates were not weighted by study sample size or precision estimates, while regional estimates are derived from country-level estimates. Consequently, these regional comparisons should be interpreted as descriptive summaries rather than precise pooled prevalence estimates. Future research would benefit from more standardized reporting and consistent methodological approaches, which would facilitate the use of formal meta-analytical techniques to generate more precise and generalizable global estimates.

## 5 Conclusions

In conclusion, this review provides a comprehensive synthesis of population-based studies from the last two decades, emphasizing variability of the global prevalence of HDP across countries and WHO regions. Substantial geographic variation in HDP prevalence was observed; however, due to heterogeneity in study design, diagnostic criteria, and the absence of harmonized population-level data, this review was not designed to identify the underlying drivers of these differences. Accordingly, the observed regional differences should be interpreted as descriptive patterns rather than evidence of specific causal or mechanistic explanations. Further, although the aggregated data highlight broad patterns and facilitate global comparisons, substantial methodological variability highlights the need for international efforts towards standardized HDP definitions and consistent screening, diagnosis, and reporting protocols. Additionally, the relative paucity and variable quality of data from low- and middle-income countries limit a comprehensive understanding of the true global burden of HDP. Future research should prioritize addressing these gaps particularly in underserved regions where the burden is high and data have historically been sparse, to improve maternal and child health outcomes worldwide.
