# Supplementary material for: Global prevalence of hypertensive disorders of pregnancy: a scoping review and global perspective by country and region
Source: Front Cardiovasc Med. 2026 Apr 22;13:1692590. doi: 10.3389/fcvm.2026.1692590 (PMC13143883; doi:10.3389/fcvm.2026.1692590)
Supplement: Supplementary file 1 [file Table1.docx]

**Appendix 1. Search Terms**

| **Terms** | **Variations of term** | **Search keywords and MeSH terms** |
| --- | --- | --- |
| Hypertension | Hypertensive, high blood pressure  Hypertens* | "hypertension"[MeSH Terms] OR hypertension[Text Word] |
| Pregnancy | Pregnant, gestation  Pregnan* | "pregnancy"[MeSH Terms] OR pregnancy[Text Word] OR gestation[Text Word] |
| Hypertension AND pregnancy |  | - "hypertension, pregnancy-induced"[MeSH Terms] OR gestational hypertension[Text Word] - "eclampsia"[MeSH Terms] OR eclampsia[Text Word] - "pre-eclampsia"[MeSH Terms] OR preeclampsia[Text Word] - ("hypertension"[MeSH Terms] OR Hypertension[Text Word]) AND ("pregnancy"[MeSH Terms] OR pregnancy[Text Word]) |
| Prevalence | Rate, incidence | "epidemiology"[Subheading] OR "prevalence"[MeSH Terms] OR prevalence[Text Word] |

**Final search strategy for PubMed:**

((("high blood pressure"[tw] OR "hypertension"[MeSH Terms] OR hypertension[tw] OR hypertensive[tw]) AND ("pregnancy"[MeSH Terms] OR pregnancy[tw] OR pregnant[tw] OR "gravidity"[MeSH Terms] OR gravidity[tw] OR gestation[tw])) OR ("eclampsia"[MeSH Terms] OR eclampsia[tw] OR "pre-eclampsia"[MeSH Terms] OR pre-eclampsia[tw] OR preeclampsia[tw])) AND ("prevalence"[MeSH Terms] OR prevalence[tw] OR "incidence"[MeSH Terms] OR incidence[tw]) AND 2004/01/01:2023/12/31[dp] Filters: English

**Final search strategy for Embase:**

(('high blood pressure':ab,ti,kw,de OR 'hypertension'/exp OR hypertension:ab,ti,kw,de OR hypertensive:ab,ti,kw,de) AND (‘pregnancy’/exp OR pregnancy:ab,ti,kw,de OR pregnant:ab,ti,kw,de OR gravidity:ab,ti,kw,de OR gestation:ab,ti,kw,de) OR (‘eclampsia’/exp OR eclampsia:ab,ti,kw,de OR ‘preeclampsia’/exp OR pre-eclampsia:ab,ti,kw,de OR preeclampsia:ab,ti,kw,de)) AND ('prevalence'/exp prevalence:ab,ti,kw,de OR 'incidence'/exp OR incidence:ab,ti,kw,de) AND [2004-2023]/py AND [english]/lim

**Appendix 2. Studies Included in the Review and Data Synthesis**

| **Study ID** | **Country** | **Title** |
| --- | --- | --- |
| Wang 2016 | Australia | Increased incidence of gestational hypertension and preeclampsia after assisted reproductive technology treatment |
| Kabir 2022 | Bangladesh | Maternal anemia and risk of adverse maternal health and birth outcomes in Bangladesh: A nationwide population-based survey |
| Souza 2020 | Brazil | Risk stratification for small for gestational age for the Brazilian population: a secondary analysis of the Birth in Brazil study |
| Nakamura-Pereira 2023 | Brazil | Agreement between maternal self-report of birth-related information and medical records in Brazil: A comparison study between public, mixed, and private hospitals |
| Liu 2011 | Canada | Incidence, risk factors, and associated complications of eclampsia |
| Baghirzada 2022 | Canada | Anesthesia-related adverse events in obstetric patients: a population-based study in Canada |
| Sabr 2022 | Canada | Severe Maternal Morbidity and Maternal Mortality Associated with Assisted Reproductive Technology |
| Ye 2014 | China | The 2011 survey on hypertensive disorders of pregnancy (HDP) in China: prevalence, risk factors, complications, pregnancy and perinatal outcomes |
| Zhan 2021 | China | Epidemiology of uterine rupture among pregnant women in China and development of a risk prediction model: analysis of data from a multicentre, cross-sectional study |
| Wang 2022 | China | Epidemiology and region-specific risk factors for low Apgar scores in China: a nationwide study |
| Sun 2023 | China | Ambient cold exposure amplifies the effect of ambient PM1 on blood pressure and hypertensive disorders of pregnancy among Chinese pregnant women: A nationwide cohort study |
| Wang 2023 | China | Risk factors combine in a complex manner in assessment for macrosomia |
| Vince 2021 | Croatia | Prevalence and impact of pre-pregnancy body mass index on pregnancy outcome: a cross-sectional study in Croatia |
| Rode 2021 | Denmark | Prediction of preterm pre-eclampsia according to NICE and ACOG criteria: descriptive study of 597,492 Danish births from 2008 to 2017 |
| Aabakke 2023 | Denmark | Risk factors for and pregnancy outcomes after SARS-CoV-2 in pregnancy according to disease severity: A nationwide cohort study with validation of the SARS-CoV-2 diagnosis |
| Jaatinen 2016 | Finland | Eclampsia in Finland; 2006 to 2010 |
| Bastola 2022 | Finland | Hypertensive disorders of pregnancy among women of migrant origin in Finland: A population-based study |
| Chen 2023 | Finland | Birth outcomes in mothers with hypertensive disorders and polycystic ovary syndrome: a population-based cohort study |
| Sesilia 2023 | Finland | The outcome of pregnancies after bariatric surgery: an observational study of pregnancies during 2004-2016 in Finland |
| Goueslard 2016 | France | Early cardiovascular events in women with a history of gestational diabetes mellitus |
| Olie 2021 | France | Prevalence of hypertensive disorders during pregnancy in France (2010-2018): The Nationwide CONCEPTION Study |
| Serrand 2021 | France | Assessment of All-Cause Cancer Incidence Among Individuals With Preeclampsia or Eclampsia During First Pregnancy |
| Martin 2022 | France | Incidence and Time Trends of Pregnancy-Related Stroke Between 2010 and 2018: The Nationwide CONCEPTION Study |
| Blacher 2023 | France | Acute coronary syndrome during pregnancy and postpartum in France: the nationwide CONCEPTION study |
| Lailler 2023 | France | Adverse Maternal and Infant Outcomes in Women With Chronic Hypertension in France (2010-2018): The Nationwide CONCEPTION Study |
| Lailler 2023 | France | Aspirin for the Prevention of Early and Severe Pre-Eclampsia Recurrence: A Real-World Population-Based Study |
| Lailler 2023 | France | Recurrence of hypertensive disorders of pregnancy: results from a nationwide prospective cohort study (CONCEPTION) |
| Lemaitre 2023 | France | Pre-gestational diabetes and the risk of congenital heart defects in the offspring: A French nationwide study |
| Mettler 2023 | France | Patterns of immunosuppressive drug use during pregnancy in women with systemic vasculitis: A nationwide population-based cohort study |
| Schneider 2012 | Germany | Gestational diabetes and preeclampsia--similar risk factor profiles? |
| Reinders 2020 | Germany | Real-world evaluation of adverse pregnancy outcomes in women with gestational diabetes mellitus in the German health care system |
| Weschenfelder 2023 | Germany | Obesity during Pregnancy and SARS-CoV-2/COVID-19-Case Series of the Registry Study "COVID-19 Related Obstetric and Neonatal Outcome Study" (CRONOS-Network) |
| Papandreou 2022 | Greece | Pre-Pregnancy Excess Weight Association with Maternal Sociodemographic, Anthropometric and Lifestyle Factors and Maternal Perinatal Outcomes |
| Papandreou 2023 | Greece | Relation of Maternal Pre-Pregnancy Factors and Childhood Asthma: A Cross-Sectional Survey in Pre-School Children Aged 2-5 Years Old |
| Pavlidou 2023 | Greece | Association of Gestational Hypertension with Sociodemographic and Anthropometric Factors, Perinatal Outcomes, Breastfeeding Practices, and Mediterranean Diet Adherence: A Cross-Sectional Study |
| Bridwell 2019 | Haiti | Hypertensive disorders in pregnancy and maternal and neonatal outcomes in Haiti: the importance of surveillance and data collection |
| Eiríksdóttir · 2015 | Iceland | Pregnancy-Induced Hypertensive Disorders before and after a National Economic Collapse: A Population Based Cohort Study |
| Singh 2018 | India | High prevalence of cesarean section births in private sector health facilities- analysis of district level household survey-4 (DLHS-4) of India |
| Chauhan 2023 | India | Burden of Anaemia, Hypertension and Diabetes among pregnant women in India |
| Grover 2023 | India | Hypertension and its correlates among pregnant women consuming tobacco in India: Findings from the National Family health Survey-4 |
| Corrigan 2021 | Ireland | Hypertension in pregnancy: Prevalence, risk factors and outcomes for women birthing in Ireland |
| Leitao 2022 | Ireland | Maternal morbidity and mortality: an iceberg phenomenon |
| Morikawa 2014 | Japan | Seasonal variation in the prevalence of pregnancy-induced hypertension in Japanese women |
| Ishikawa 2023 | Japan | Risk of major congenital malformations associated with first-trimester antihypertensives, including amlodipine and methyldopa: A large claims database study 2010-2019 |
| Khader 2018 | Jordan | Preeclampsia in Jordan: incidence, risk factors, and its associated maternal and neonatal outcomes |
| Schaap 2019 | Netherlands | A national surveillance approach to monitor incidence of eclampsia: The Netherlands Obstetric Surveillance System |
| vanZijl 2020 | Netherlands | Trends in preterm birth in singleton and multiple gestations in the Netherlands 2008-2015: A population-based study |
| Rasmussen 2014 | Norway | Maternal obesity and excess of fetal growth in pre-eclampsia |
| Johnson 2016 | Botswana | Hypertensive disease in pregnancy in Botswana: Prevalence and impact on perinatal outcomes |
| Nurgaliyeva 2020 | Kazkhstan | Epidemiology of pre-eclampsia in the Republic of Kazakhstan: Maternal and neonatal outcomes |
| Maducolil 2021 | Qatar | Preeclampsia: incidence, determinants, and pregnancy outcomes from maternity hospitals in Qatar: a population-based case-control study |
| Al-Obaidly 2022 | Qatar | Perinatal outcomes of intrahepatic cholestasis of pregnancy from two birth cohorts: A population-based study |
| Lucovnik 2020 | Slovenia | Changes in perinatal outcomes after implementation of IADPSG criteria for screening and diagnosis of gestational diabetes mellitus: A national survey |
| Verschueren 2020 | Suriname | Why magnesium sulfate 'coverage' only is not enough to reduce eclampsia: Lessons learned in a middle-income country |
| Sultan 2016 | Sweden | Development and validation of risk prediction model for venous thromboembolism in postpartum women: Multinational cohort study |
| Liljestrom 2018 | Sweden | Obstetric emergencies as antecedents to neonatal hypoxic ischemic encephalopathy, does parity matter? |
| Mantel 2020 | Sweden | Association of Maternal Eating Disorders With Pregnancy and Neonatal Outcomes |
| Byberg 2021 | Sweden | Preeclampsia and risk of early-childhood asthma: a register study with sibling comparison and an exploration of intermediate variables |
| Yang 2021 | Sweden | Preeclampsia Prevalence, Risk Factors, and Pregnancy Outcomes in Sweden and China |
| Beer 2022 | Sweden | Associations of preterm birth, small-for-gestational age, preeclampsia and placental abruption with attention-deficit/hyperactivity disorder in the offspring: Nationwide cohort and sibling-controlled studies |
| Byberg 2022 | Sweden | Preeclampsia and risk of early-childhood asthma: a register study with sibling comparison and an exploration of intermediate variables |
| Dickmark 2022 | Sweden | Risk factors for seizures in the vigorous term neonate: A population-based register study of singleton births in Sweden |
| Chang 2010 | Taiwan | Working hours and risk of gestational hypertension and pre-eclampsia |
| Huang 2016 | Taiwan | A nationwide population analysis of antenatal and perinatal complications among nurses and nonmedical working women |
| Sun 2019 | Taiwan | Trends and risk factors of stillbirth in Taiwan 2006-2013: a population-based study |
| Yu 2020 | Taiwan | Does sex matter? Association of fetal sex and parental age with pregnancy outcomes in Taiwan: a cohort study |
| Chen 2021 | Taiwan | Childhood neurodevelopmental disorders and maternal hypertensive disorders of pregnancy |
| Huang 2022 | Taiwan | Effect of maternal hypertensive disorders during pregnancy on offspring's early childhood body weight: A population-based cohort study |
| Wu 2022 | Taiwan | Increased risk of early-onset childhood systemic lupus erythematosus for children born to affected parents: A nationwide child-parent cohort study |
| Chen 2023 | Taiwan | Childhood neurodevelopmental disorders and maternal diabetes: A population-based cohort study |
| Wang 2023 | Taiwan | Maternal hypertensive pregnancy disorders increase childhood intellectual disability hazards independently from preterm birth and small for gestational age |
| Lim 2019 | South Korea | Effects of central obesity on maternal complications in Korean women of reproductive age |
| Noh 2020 | South Korea | A cohort study of antihypertensive use during pregnancy in South Korea, 2013-2017 |
| Kim 2021 | South Korea | Risk factors associated with idiopathic sudden sensorineural hearing loss in pregnant South Korean women: a nationwide population-based study |
| Cho 2022 | South Korea | Prior pregnancy complications and maternal cardiovascular disease in young Korean women within 10 years after pregnancy |
| Kim 2022 | South Korea | Impact of Maternal Age on Singleton Pregnancy Outcomes in Primiparous Women in South Korea |
| Kim 2022 | South Korea | Seasonal variations in the occurrence of preeclampsia and potential implication of upper respiratory infections in South Korea |
| Kim 2022 | South Korea | Korea hypertension fact sheet 2021: analysis of nationwide population-based data with special focus on hypertension in women |
| Kim 2022 | South Korea | Risk factors associated with idiopathic sudden sensorineural hearing loss in pregnant South Korean women: a nationwide population-based study |
| Kim 2022 | South Korea | Maternal and Neonatal Risk Factors Affecting the Occurrence of Neurodevelopmental Disorders: A Population-Based Nationwide Study |
| Park 2022 | South Korea | Increased Cardiac Arrhythmia After Pregnancy-Induced Hypertension: A South Korean Nationwide Database Study |
| Park 2022 | South Korea | The Risk Factors, Incidence and Prognosis of Postpartum Breast Cancer: A Nationwide Study by the SMARTSHIP Group |
| Bae 2023 | South Korea | Incidence and Risk Factors for Pregnancy-Related de Quervain's Tenosynovitis in South Korea: A Population-Based Epidemiologic Study |
| Kim 2023 | South Korea | Differential trend of mild and severe preeclampsia among nulliparous women: a population-based study of South Korea |
| Lee 2023 | South Korea | Risk of retinopathy in women with pregnancy-induced hypertension: a nationwide population-based cohort study of 9-year follow-up after delivery |
| Lee 2023 | South Korea | Long term renal outcome after hypertensive disease during pregnancy: a nationwide population-based study |
| Lee 2023 | South Korea | Impact of moderate-to-late preterm birth on neurodevelopmental outcomes in young children: Results from retrospective longitudinal follow-up with nationally representative data |
| Lee 2023 | South Korea | Metabolic dysfunction-associated fatty liver disease as a risk factor for adverse outcomes in subsequent pregnancy: a nationwide cohort study |
| Nam 2023 | South Korea | Headaches during pregnancy and the risk of subsequent stroke |
| Shim 2023 | South Korea | Risk of adverse obstetric outcomes in patients with a history of endometrial cancer: A nationwide population-based cohort study |
| Lopez-de-Andres 2020 | Spain | A Population-Based Study of Diabetes During Pregnancy in Spain (2009-2015): Trends in Incidence, Obstetric Interventions, and Pregnancy Outcomes |
| Knight 2007 | United Kingdom | Eclampsia in the United Kingdom 2005 |
| Kayem 2011 | United Kingdom | Maternal and obstetric factors associated with delayed postpartum eclampsia: a national study population |
| Khandwala 2018 | United States | Association of paternal age with perinatal outcomes between 2007 and 2016 in the United States: population based cohort study |
| Rossi 2019 | United States | Predictive Model of Factors Associated With Maternal Intensive Care Unit Admission |
| Tripathi 2019 | United States | Trends in Incidence and Outcomes of Pregnancy-Related Acute Myocardial Infarction (From a Nationwide Inpatient Sample Database) |
| DeSisto 2021 | United States | Hypertension at delivery hospitalization - United States, 2016-2017 |
| Passarella 2021 | United States | Maternal and fetal outcomes in pregnancies with obstructive sleep apnea |
| Rozario 2021 | United States | The additive effect of interpregnancy interval and maternal body mass index on pregnancy induced hypertension in the U.S |
| Xiao 2021 | United States | Trends in eclampsia in the United States, 2009-2017: a population-based study |
| Cameron 2022 | United States | Trends in the Incidence of New-Onset Hypertensive Disorders of Pregnancy Among Rural and Urban Areas in the United States, 2007 to 2019 |
| Chen 2022 | United States | Hepatitis C is associated with more adverse pregnancy outcomes than hepatitis B: A 7-year national inpatient sample study |
| Feferkorn 2022 | United States | The relation between cigarette smoking with delivery outcomes. An evaluation of a database of more than nine million deliveries |
| Felske 2022 | United States | Comparing adverse neonatal and maternal outcomes of chlamydia, gonorrhoea, and syphilis infections and co-infections in pregnancy |
| Ford 2022 | United States | Hypertensive Disorders in Pregnancy and Mortality at Delivery Hospitalization - United States, 2017-2019 |
| Freaney 2022 | United States | Temporal Trends in Adverse Pregnancy Outcomes in Birthing Individuals Aged 15 to 44 Years in the United States, 2007 to 2019 |
| Gambahaya 2022 | United States | Racial Differences in Delivery Outcomes Among Women With Peripartum Cardiomyopathy |
| Guglielminotti 2022 | United States | Nurse workforce diversity and reduced risk of severe adverse maternal outcomes |
| Huang 2022 | United States | Pregnancies With Cirrhosis Are Rising and Associated With Adverse Maternal and Perinatal Outcomes |
| Kim 2022 | United States | Weekend delivery and maternal-neonatal adverse outcomes in low-risk pregnancies in the United States: A population-based analysis of 3-million live births |
| Kloppenburg 2022 | United States | Prevalence and outcomes of hypertension in pregnancy in non-metropolitan and metropolitan communities |
| Mazza 2022 | United States | Association of Pregnancy Characteristics and Maternal Mortality With Amniotic Fluid Embolism |
| McLaren 2022 | United States | Change in prevalence of chronic hypertension in pregnancy after the updated ACC/AHA hypertension guidelines |
| Shen 2022 | United States | Disparities in Adverse Maternal Outcomes Among Five Race and Ethnicity Groups |
| Tarar 2022 | United States | A national study of pregnancy-related maternal and fetal outcomes in women with inflammatory bowel disease |
| Thakkar 2022 | United States | Temporal trends of arrhythmias at delivery hospitalizations in the United States: Analysis from the National Inpatient Sample, 2009-2019 |
| Wang 2022 | United States | Association of Preterm Singleton Birth With Fertility Treatment in the US |
| Wen 2022 | United States | Trends and outcomes for deliveries with hypertensive disorders of pregnancy from 2000 to 2018: A repeated cross-sectional study |
| Wheeler 2022 | United States | Estimated Prevalence of Risk Factors for Preeclampsia Among Individuals Giving Birth in the US in 2019 |
| Wilson 2022 | United States | The association between narcolepsy during pregnancy and maternal-fetal risk factors/outcomes |
| Zahid 2022 | United States | Trends, Predictors, and Outcomes of Cardiovascular Complications at Delivery Associated With Gestational Diabetes: A National Inpatient Sample Analysis (2004-2019) |
| Azad 2023 | United States | Peripartum cardiomyopathy delivery hospitalization and postpartum readmission trends, risk factors, and outcomes |
| Christopher 2023 | United States | Stroke-related risk factors during pregnancy in women who underwent metabolic and bariatric surgery compared with women who have not undergone metabolic and bariatric surgery |
| Diab 2023 | United States | Temporal Trend in Maternal Morbidity and Comorbidity |
| Fink 2023 | United States | Trends in Maternal Mortality and Severe Maternal Morbidity during Delivery-Related Hospitalizations in the United States, 2008 to 2021 |
| Fronek 2023 | United States | Prevalence of hypertensive disorders of pregnancy at or beyond 39 weeks gestational age and associated maternal complications |
| Goins 2023 | United States | Maternal and neonatal outcomes in patients with hepatitis C and intrahepatic cholestasis of pregnancy: The sum of the parts |
| Green 2023 | United States | Trends, Characteristics, and Maternal Morbidity Associated With Unhoused Status in Pregnancy |
| Huang 2023 | United States | Risk of delivery complications among pregnant people experiencing housing insecurity |
| Kilani 2023 | United States | Autoimmune Hepatitis and Obstetrical Outcomes: A Nationwide Assessment |
| Lisonkova 2023 | United States | Early coronavirus disease 2019 restrictive measures and changes in maternal characteristics, use of assisted reproductive technology, and stillbirth |
| Majmundar 2023 | United States | Prevalence, trends, and outcomes of cardiovascular diseases in pregnant patients in the USA: 2010-19 |
| Markson 2023 | United States | Trends in the incidence and in-patient outcomes of acute myocardial infarction in pregnancy: Insights from the national inpatient sample |
| Mogos 2023 | United States | Pregnancy-associated takotsubo cardiomyopathy hospitalizations in the United States |
| Niu 2023 | United States | Maternal and prenatal outcomes of hemochromatosis in pregnancy: A population-based study |
| Niu 2023 | United States | Maternal and fetal outcomes of acute pancreatitis in pregnancy: a population-based study |
| Raina 2023 | United States | The effect of maternal hypertension and maternal mental illness on adverse neonatal outcomes: A mediation and moderation analysis in a U.S. cohort of 9 million pregnancies |
| Sorokine 2023 | United States | Maternal and neonatal outcomes in women with a history of chemotherapy exposure: a population-based study of 8 million obstetric admissions |
| Sun 2023 | United States | Dose-response association between maternal smoking during pregnancy and the risk of infant death: a nationwide, population-based, retrospective cohort study |
| Wang 2023 | United States | Eclampsia, in vitro fertilization and the risk of preterm birth: a retrospective cohort study based on 2,880,759 samples |
| Warner 2023 | United States | Trends in cardiovascular complications of pregnancy: A nationwide inpatient sample analysis |
| Zahid 2023 | United States | Cardiovascular Complications During Delivery Admissions Associated With Assisted Reproductive Technology (from a National Inpatient Sample Analysis 2008 to 2019) |
| Briozzo 2023 | Uruguay | COVID-19 mitigation measures increase preterm birth and low birth weight in the public healthcare system in Uruguay |

**Supplemental Table 1. Quality Assessment Scale Criteria**

| **Quality Assessment Domain** | **Score = 1** | **Score = 0** |
| --- | --- | --- |
| Cohort Selection Strategy | Random, systematic, stratified, or cluster sampling | Convenience sampling or unspecified |
| Sample size | Sample size  (final analytic n) $\geq$ 2500 | Sample size  (final analytic n) < 2500 |
| Assessment of HDP | Healthcare professional diagnosed in a healthcare setting (medical records), or diagnosed in healthcare setting or in the field with two separate blood pressure measurements 4 hours apart, or extracted from birth certificates/surveillance record | Unspecified |
| Age-standardized HDP Prevalence | Age-standardized rates were reported | Only crude rates were reported |
| Types of HDP | Only one HDP subtype or multiple individual HDP subtypes | Composite HDP with≥2 subtypes |
| **Composite score:**  **0–1 = Poor Quality**  **2–4 = Moderate Quality**  **5 = Good Quality** | | |

**Abbreviations:** HDP, hypertensive disorders of pregnancy

**Supplemental Table 2: Hypertensive Disorders of Pregnancy Definition Used per Study**

| **Study ID** | **Country** | **How was HDP defined?** |
| --- | --- | --- |
| Wang 2016 | Australia | \| GHTN: blood pressure recording of more than 140/90 mm Hg on at least two occasions more than 6 hours apart without evidence of chronic hypertension after 20 weeks' gestation. \| \| --- \| \| PE: presence of hypertension with proteinuria, maternal organ dysfunction, or uteroplacental dysfunction. \| |
| Kabir 2022 | Bangladesh | Hypertension (SBP >=140 mmHg/ DBP>=90 mmHg OR taking antihypertensive medication. |
| Souza 2020 | Brazil | None provided. |
| Nakamura-Pereira 2023 | Brazil | None provided. |
| Liu 2011 | Canada | Eclampsia: development of convulsions during pregnancy or in the postpartum period in women with signs and symptoms of preeclampsia, or gestational or pre-existing hypertension, identified by ICD-10 CA codes (O15.0-O15.2 or O15.9). |
| Baghirzada 2022 | Canada | HDP: gestational hypertension, eclampsia, preeclampsia. |
| Sabr 2022 | Canada | HDP: severe hypertensive complications, including severe preeclampsia, HELLP syndrome, or eclampsia |
| Ye 2014 | China | \| HDP: occurrence in the period between antepartum and postpartum. According to the latest version of classification system by the National High Blood Pressure Education Program (NHBPEP). \| \| --- \| \| GHTN: systolic blood pressure (SBP) of >=140 mmHg and/or diastolic blood pressure (DBP) of >=90 mmHg without proteinuria, which developed after 20 weeks of gestation and returned to normal within 12 weeks of postpartum. \| |
| Zhan 2021 | China | None provided. |
| Wang 2022 | China | HDP: gestational hypertension, preeclampsia/eclampsia, chronic hypertension, and chronic hypertension superimposed by preeclampsia/eclampsia. |
| Sun 2023 | China | \| GHTN: new-onset hypertension (mean SBP 140 mmHg or DBP 90 mmHg) after 20 weeks of gestation \| \| --- \| \| Preeclampsia: hypertension (SBP/DBP 140/90 mmHg) and proteinuria of 1+ or more on a dipstick test or the protein level in the urine 300 mg/24-h after 20 weeks of gestation; or hypertension plus the involvement of one organ or system in women with previous normal BP. \| |
| Wang 2023 | China | \| GHTN: systolic blood pressure 140 mmHg or a diastolic blood pressure 90 mmHg, or both, on two occasions at least 4 h apart after 20 gestational weeks, but without proteinuria, in a woman with a previously normal blood pressure. \| \| --- \| \| Preeclampsia: systolic blood pressure 140 mmHg or diastolic blood pressure 90 mmHg, measured on at least two occasions after 20 weeks with proteinuria. In the absence of proteinuria, preeclampsia is diagnosed as hypertension in association with thrombocytopenic hemorrhage, cerebral arterial ischemia, infarction, or drug use. \| |
| Vince 2021 | Croatia | None provided. |
| Rode 2021 | Denmark | \| PE: gestational hypertension accompanied by proteinuria and/or specific signs or symptoms of significant end-organ dysfunction. \| \| --- \| \| GHTN: blood pressure140 mmHg systolic or 90 mmHg diastolic on three separate readings after 20 weeks gestation in a woman with previously normal blood pressure. \| |
| Aabakke 2023 | Denmark | \| GHTN: ICD-10 code O13. \| \| --- \| \| Preeclampsia: ICD-10 code O14. \| \| Eclampsia: ICD-10 code O15. \| |
| Jaatinen 2016 | Finland | \| Eclampsia: occurrence of one or more seizures before, during or after delivery in women with signs or symptoms of preeclampsia. \| \| --- \| \| Preeclampsia: systolic blood pressure was repeatedly 140 mmHg and/or the diastolic blood pressure was repeatedly 90 mmHg after 20 weeks of gestation in association with proteinuria. \| |
| Bastola 2022 | Finland | GHTN: systolic blood pressure of 140mmHg or the diastolic blood pressure of 90mmHg identified after 20weeks gestation. |
| Chen 2023 | Finland | \| HDP: chronic hypertension, gestational hypertension, and pre-eclampsia \| \| --- \| \| CHTN: blood pressure (BP) 140/ 90 mmHg predating pregnancy or diagnosed before 20 weeks gestation, identified based on ICD-10: I10I13, and O10. \| \| GHTN: is BP 140/90 mmHg arising de novo after 20 weeks of gestation without proteinuria and biochemical or hematological abnormalities, identified based on ICD-10: O13. \| \| Preeclampsia: new-onset or chronic hypertension combined with proteinuria at 20 weeks or more of gestation, identified according to ICD-10: O11, and O14 (Supplementary Table S1). \| |
| Sesilia 2023 | Finland | None provided. |
| Goueslard 2016 | France | Hypertensive disorders of pregnancy: ICD-10 code O16. |
| Olie 2021 | France | \| GHTN: hospitalization with a primary diagnosis of gestational hypertension during pregnancy (ICD-10 code: O13) or at least one delivery of antihypertensive medication between 20 weeks of gestation and 6 weeks postpartum. \| \| --- \| \| Preeclampsia, HELLP syndrome, and eclampsia were identified by a hospitalization with a diagnosis of preeclampsia (O14), HELLP syndrome (O14.2), or eclampsia (O15). \| |
| Serrand 2021 | France | \| Eclampsia: ICD-10 code O15. \| \| --- \| \| Preeclampsia: ICD-10 codes O11 and O14. \| |
| Martin 2022 | France | \| CHTN: identified by at least 3 deliveries of antihypertensive drugs on different dates over a 12-month period or on 2 dates if at least 1 large pack (90 pills) of antihypertensive drugs was dispensed. \| \| --- \| \| HDP: identified using an algorithm based on hospitalizations for pre-eclampsia (O14), hemolysis, elevated liver enzymes, low platelet count (HELLP) syndrome (O14.2), or eclampsia (O15). In women without pre-existing chronic hypertension, gestational hypertension was identified in our database by a hospitalization with a primary diagnosis of gestational hypertension during pregnancy (O13) or at least 1 delivery of antihypertensive medication between 20 weeks of gestation and 6 weeks of postpartum without differential diagnosis of preterm labor. \| |
| Blacher 2023 | France | \| CHTN: dispensing of antihypertensive or antidiabetic medications on at least 3 occasions in the year preceding pregnancy, or on 2 occasions if at least 1 large package (90 pills) of drugs was dispensed. \| \| --- \| \| PE: ICD-10 code O14. \| \| Eclampsia: ICD-10 code O15. \| \| GHTN: identified in women without preexisting chronic hypertension, either through hospitalization with a primary diagnosis of GH during pregnancy (O13), or through the dispensing of antihypertensive medication at least once between 20 weeks of gestation and 6 weeks postpartum. \| |
| Lailler 2023 | France | CHTN: hypertension predating pregnancy or diagnosed before 20 weeks of gestation; ICD-10 codes: 010, O12. |
| Lailler 2023 | France | \| CHTN: dispensing of antihypertensive drugs on at least three different dates (two different dates if at least one large package of 90 pills of antihypertensive drugs was dispensed) between 1year preceding the first pregnancy and 20 weeks of gestation. \| \| --- \| \| GHTN: dispensing of at least one antihypertensive drug between 20 weeks of gestation and 6 weeks postpartum, or a hospital stay with a diagnosis of GH (ICD-10 code O13), in the absence of previous chronic hypertension. \| \| Severe preeclampsia: ICD-10 code 014.1. \| \| Eclampsia: ICD-10 code O15. \| |
| Lailler 2023 | France | \| CHTN: dispensing of antihypertensive drugs on at least three different dates (two different dates if at least one large package of 90 pills of antihypertensive drugs was dispensed) between 1year preceding the first pregnancy and 20weeks of gestation. \| \| --- \| \| GHTN: dispensing of at least one antihypertensive drug between 20 weeks of gestation and 6weeks postpartum, or a hospital stay with a diagnosis of GH (ICD-10 code O13), in the absence of previous chronic hypertension. \| \| Severe preeclampsia: ICD-10 code 014.1. \| \| Eclampsia: ICD-10 code O15. \| |
| Lemaitre 2023 | France | Preeclampsia: defined as the combination of systolic blood pressure > 140 mmHg or diastolic blood pressure >90 mmHg and proteinuria. |
| Mettler 2023 | France | \| Pre-existing hypertension complicating pregnancy, childbirth and the puerperium: ICD-10 code 010. \| \| --- \| \| Pre-existing hypertension with pre-eclampsia: ICD-10 code O11. \| \| Gestational edema and proteinuria without hypertension: ICD-10 code O12. \| \| Gestational hypertension without significant proteinuria: ICD-10 code O13. \| \| Preeclampsia: ICD-10 code O14. \| \| Eclampsia: ICD-10 code O15. \| |
| Schneider 2012 | Germany | Preeclampsia: pregnancies with a simultaneous diagnosis of hypertension (repeatedlyN140/90) and proteinuria. |
| Reinders 2020 | Germany | \| CHTN: ICD-10 code O10. \| \| --- \| \| GHTN: ICD-10 code O13. \| \| Preeclampsia: ICD-10 code O14. \| \| Eclampsia: ICD-10 code O15. \| |
| Weschenfelder 2023 | Germany | None provided. |
| Papandreou 2022 | Greece | None provided. |
| Papandreou 2023 | Greece | None provided. |
| Pavlidou 2023 | Greece | GHTN: classified as systolic blood pressure >140 mmHg for average of three readings. |
| Bridwell 2019 | Haiti | \| GHTN: (systolic blood pressure (SBP) level 140 mmHg or diastolic blood pressure (DBP) 90 mmHg on at least two occasions, four or more hours apart after 20 weeks of gestation). \| \| --- \| \| Preeclampsia: SBP level of 140 mmHg or DBP 90 mmHg (on at least two occasions, four or more hours apart) with proteinuria (300 mg/24 h) in at least 2 urine specimens collected 6 hours or more apart Eclampsia: SBP level of 140 mmHg or DBP 90 mmHg, proteinuria, and seizures. \| \| GHTN: (systolic blood pressure (SBP) level 140 mmHg or diastolic blood pressure (DBP) 90 mmHg on at least two occasions, four or more hours apart after 20 weeks of gestation). \| \| Preeclampsia: SBP level of 140 mmHg or DBP 90 mmHg (on at least two occasions, four or more hours apart) with proteinuria (300 mg/24 h) in at least 2 urine specimens collected 6 hours or more apart Eclampsia: SBP level of 140 mmHg or DBP 90 mmHg, proteinuria, and seizures. \| |
| Eiríksdóttir · 2015 | Iceland | \| GHTN: ICD-10 code O13, defined as newly diagnosed hypertension (SBP 140 mmHg; DBP 90 mmHg) after 20 weeks of gestation. \| \| --- \| \| Preeclampsia: pre-existing or gestational hypertension concurring with significant proteinuria (>300 mg protein in 24-hour urine sample). In the present study, women were classified as having preeclampsia if they had been diagnosed with pre-existing hypertensive disorder with superimposed preeclampsia [ICD-10 code O11], preeclampsia [ICD-10 code O14], or eclampsia [ICD-10 code O15]. \| |
| Singh 2018 | India | None provided. |
| Chauhan 2023 | India | Hypertension: A pregnant woman is classified as having hypertension if she has SBP 140 mmHg. |
| Grover 2023 | India | Hypertensive: SBP > 140 mmHg or DBP > 90 mmHg in the average of three measurements. |
| Corrigan 2021 | Ireland | \| GHTN (ICD-10 codes O13 and O16). \| \| --- \| \| PE (ICD-10 codes O13; O14.0 and O14.9). \| \| Severe PE (ICD-10 codes O14.1). \| \| Eclampsia (ICD-10 codes O15.0, O15.1, O15.2 and O15.9). \| \| HELLP syn-drome (ICD-10 codes O14.2). \| |
| Leitao 2022 | Ireland | \| GHTN: O12, O13. \| \| --- \| \| Preeclampsia: O14. \| \| Eclampsia: O15. \| \| Preexisting hypertension: ICD-10 code O10. \| \| Preexisting hypertension with preeclampsia: O11. \| |
| Morikawa 2014 | Japan | PIH: including gestational hypertension and pre-eclampsia. |
| Ishikawa 2023 | Japan | Hypertensive diseases (ICD-10 codes I10 I15) diagnosed before the end of the first trimester, including the pre-pregnancy period; or (ii) edema, proteinuria, and hypertensive disorders in pregnancy, childbirth, and the puerperium (O10 -O16), excluding gestational edema, and proteinuria without hypertension (O12) due to the nature of the disease without hypertension, diagnosed during the first trimester. |
| Khader 2018 | Jordan | Preeclampsia was defined according to International Society for the Study of Hypertension in Pregnancy (ISSHP). |
| Schaap 2019 | Netherlands | \| Since there was no international consensus definition for eclampsia at the start of this registration, we purposefully used a broad definition for case ascertainment in order to facilitate comparisons \| \| --- \| \| with women from both the previous LEMMoN registration and the United Kingdom Obstetric Surveillance System (UKOSS) (Supporting Information Table S2). \| |
| vanZijl 2020 | Netherlands | None provided. |
| Rasmussen 2014 | Norway | In Norway, the diagnosis of preeclampsia is in accordance with the recommendations of the American College of Obstetricians and Gynecologists, which defined preeclampsia as the presence of systolic blood pressure of 140 mmHg or a diastolic pressure of 90 mmHg on at least two occasions 6 hours apart after 20 weeks of gestation with proteinuria defined as excretion of 0.3 g/day, equivalent to at least 1+ on a urine reagent strip. |
| Johnson 2016 | Botswana | \| HTN: at least one systolic BP (SBP) > 140 or diastolic BP (DBP) > 90. \| \| --- \| \| Severe hypertension: SBP >160 or DBP>110 during pregnancy. \| |
| Nurgaliyeva 2020 | Kazkhstan | PE: new onset hypertension (> 140 mmHg systolic and/or > 90 mmHg diastolic blood pressure) after 20 weeks of gestation and proteinuria and/or progressive maternal acute kidney failure, impaired liver function, neuro-logical disturbances, hemolysis or thrombocytopenia, and/or fetal growth restriction. |
| Maducolil 2021 | Qatar | PE: diagnosed based on ACOG definition criteria based on a task force report in 2013. |
| Al-Obaidly 2022 | Qatar | PE: new onset of hypertension and proteinuria or of hypertension and significant end-organ dysfunction with or without proteinuria in the last half of pregnancy or postpartum. |
| Lucovnik 2020 | Slovenia | None provided. |
| Verschueren 2020 | Suriname | Eclampsia: defined using the Delphi-validated International Network of Obstetric Surveillance System (INOSS) [6], as seizure(s) in a woman during pregnancy or up to 14 days postpartum, without any other attributable cause, and with at least one of the following conditions: Hypertension (140 mmHg systolic or 90 mmHg diastolic) Proteinuria (at least 1 g/l] on dipstick testing). |
| Sultan 2016 | Sweden | \| CHTN: ICD-10 code O10. \| \| --- \| \| GHTN: ICD-10 code O13. \| \| PE/E: ICD-10 codes O14 and O15. \| |
| Liljestrom 2018 | Sweden | HDPs: included chronic or gestational hypertension, preeclampsia, and/or eclampsia, and diabetes mellitus Labor dystocia included primary and secondary dystocia, as well as a prolonged second stage. Data on hypertensive disorders, diabetes mellitus, premature rupture of membranes, labor dystocia, fetal-pelvic disproportion, meconium-stained amniotic fluid, fever during delivery, clinical chorioamnionitis, and failed vacuum extraction or forceps were identified by diagnostic ICD-10 codes (see Supplementary material, Table S1). |
| Mantel 2020 | Sweden | Preeclampsia: ICD-10 codes O14 and O15. |
| Byberg 2021 | Sweden | \| PE: divided into mild/moderate and severe forms (based on International Classification of Diseases, ICD-10 diagnoses from the NPR, O14.0, O14.9). \| \| --- \| \| PE with preexisting hypertension: ICD-10 code O11.9. \| \| Eclampsia: ICD-10 codes O15.0, O15.1, O15.2 and O15.9. \| |
| Yang 2021 | Sweden | \| GHTN: ICD-10 code O13. \| \| --- \| \| Mild PE: ICD-10c ode O14. \| \| Severe PE: ICD-10 codes O14.1 and O14.9. \| \| E: ICD-10 code O15. \| |
| Beer 2022 | Sweden | PE: ICD-10 code O14, classified as term (37 weeks) or preterm (34-36 or <34 weeks) |
| Byberg 2022 | Sweden | PE: characterized by increased blood pressure and proteinuria, and increased risk of morbidity and mortality in both mother and child, divided into mild/moderate and severe forms (based on International Classification of Diseases, ICD-10 diagnoses from the NPR code O14). |
| Dickmark 2022 | Sweden | \| CHTN: ICD-10 code O10. \| \| --- \| \| GHTN: ICD-10 code O13.9.0. \| \| PE: ICD-10 code O14. \| \| E: ICD-10 code O15. \| |
| Chang 2010 | Taiwan | \| GHTN: systolic blood pressure of at least 140 mmHg or a diastolic blood pressure of at least 90 mmHg on at least two occasions taken at least 6h after 20 weeks of gestation in women who previously had normal blood pressure. \| \| --- \| \| PE: defined as the presence of gestational hypertension with proteinuria. \| |
| Huang 2016 | Taiwan | PIH and PE: ICD-10 codes 642.0, 642.1-642.5, 642.7, 642.9, or 760.0. |
| Sun 2019 | Taiwan | None provided. |
| Yu 2020 | Taiwan | \| GHTN/PE: ICD-9-CM: 642. \| \| --- \| \| E: ICD-9-CM: 642.6. \| |
| Chen 2021 | Taiwan | \| CHTN: ICD-9 401-405. \| \| --- \| \| PIH/PE: ICD-9 642 during each pregnancy after 20 weeks of gestation Mothers who were diagnosed with hypertension disorders before pregnancy (in the previous 3y), before 20 weeks of gestation, or whose hypertension diagnosis persisted 12 weeks postpartum were deemed to have chronic hypertension, regardless of whether they had PIH/preeclampsia. \| |
| Huang 2022 | Taiwan | None provided. |
| Wu 2022 | Taiwan | PIH/PE/E: ICD-10 codes O13, O16, O14, O15. |
| Chen 2023 | Taiwan | \| CHTN: ICD-9: 401-405. \| \| --- \| \| GHTN/PE: ICD-9 642.3-7. \| |
| Wang 2023 | Taiwan | \| CHTN: 140/90 mmHg before pregnancy or during the first 20 weeks of gestation, while hypertension arising de novo at or after 20 weeks included gestational hypertension (absence of proteinuria and without biochemical or hematological abnormalities). \| \| --- \| \| PE: complicated with proteinuria or evidence of maternal acute kidney injury, liver dysfunction, neurological features, hemolysis or thrombocytopenia). \| |
| Lim 2019 | South Korea | Hypertension: systolic blood pressure/diastolic blood pressure >140/90mmHg or at least 1 claim per year for an antihypertensive medication prescription under ICD-10 codes I10-I15. |
| Noh 2020 | South Korea | \| GHTN: ICD-10 code O13. \| \| --- \| \| Preeclampsia: ICD-10 code O14. \| \| Eclampsia: ICD-10 code O15. \| |
| Kim 2021 | South Korea | None provided. |
| Cho 2022 | South Korea | \| PE superimposed on CHTN: ICD-10 code O13. \| \| --- \| \| GHTN: ICD-10 code O14. \| \| PE: ICD-10 code O15. \| \| E: ICD-10 code O16. \| |
| Kim 2022 | South Korea | PE: ICD-10 codes O11, O14, and O15. |
| Kim 2022 | South Korea | PE: ICD-10 codes O11, O14, and O15. |
| Kim 2022 | South Korea | PE: ICD-10 codes O11, O14, and O15. |
| Kim 2022 | South Korea | PE: ICD-10 codes O11, O14, and O15. |
| Kim 2022 | South Korea | \| GHTN without significant proteinuria: ICD-10 code O13. \| \| --- \| \| PE: ICD-10 code O14. \| \| E: ICD-10 code O15. \| |
| Park 2022 | South Korea | GHTN: ICD-10 code O13 Preeclampsia: ICD-10 code O14 Eclampsia: ICD-10 code O15. |
| Park 2022 | South Korea | GHTN: ICD-10 code O13 Preeclampsia: ICD-10 code O14 Eclampsia: ICD-10 code O15. |
| Bae 2023 | South Korea | \| HDP: ICD-10 code O11. \| \| --- \| \| PE superimposed on CHTN: ICD-10 code O13. \| \| GHTN: ICD-10 code O14. \| \| PE: ICD-10 code O15. \| \| E: ICD-10 code O16. \| |
| Kim 2023 | South Korea | PE: ICD-10 codes O11, O14, and O15. |
| Lee 2023 | South Korea | None provided. |
| Lee 2023 | South Korea | \| GHTN: ICD-10 code O13. \| \| --- \| \| Preeclampsia: ICD-10 code O14. \| \| Superimposed preeclampsia ICD-10 code O11. \| \| Mild to moderate preeclampsia: ICD-10 code O14.0. \| \| Severe preeclampsia: ICD-10 code O14.1. \| \| HELLP syndrome: ICD-10 code O14.2 \| \| Preeclampsia, unspecified: ICD-10 code O14.9. \| \| Eclampsia: ICD-10 code O15. \| \| Convulsions following conditions in O10-O14 and O16. \| |
| Lee 2023 | South Korea | GHTN: ICD-10 code O13. |
| Lee 2023 | South Korea | \| GHTN: ICD-10 code O13. \| \| --- \| \| Preeclampsia: ICD-10 code O14. \| \| Eclampsia: ICD-10 code O15. \| |
| Nam 2023 | South Korea | HDP: chronic hypertension, gestational hypertension (include pre-eclampsia and eclampsia). |
| Shim 2023 | South Korea | None provided. |
| Lopez-de-Andres 2020 | Spain | None provided. |
| Knight 2007 | United Kingdom | E: convulsion(s) during pregnancy or in the first 10 days postpartum, together with at least two of the following features within 24 hours of the convulsion(s): Hypertension (DBP 90 mmHg, a maximum diastolic of 90 mmHg and a diastolic increment of 25 mmHg); Proteinuria (at least 1protein in a random urine sample or 0.3 g in a 24-hour collection). d) Thrombocytopenia (platelet count of less than 1003109/L); raised plasma alanine aminotransferase concentration (42 iu/l) or an increased plasma aspartate aminotransferase concentration (42 IU/L). |
| Kayem 2011 | United Kingdom | E: any woman with convulsion(s) during pregnancy or in the first 28 days postpartum, together with at least two of the following features within 24 hours of the convulsion(s): (a) hypertension (a booking diastolic pressure of <90mmHg, a maximum diastolic pressure of > 90mmHg and a diastolic increment of >25mmHg); (b) proteinuria (at least 1+ protein in a random urine sample or >0.3g/dl in a 24 hour collection); (c) thrombocytopenia (platelet count of less than 100 Ã— 109/l); and (d) raised plasma alanine aminotransferase (ALT) concentration (>42 IU/l) or an increased plasma aspartate aminotransferase concentration (>42 IU/l) |
| Khandwala 2018 | United States | None provided. |
| Rossi 2019 | United States | None provided. |
| Tripathi 2019 | United States | \| GHTN: ICD-9 code O13. \| \| --- \| \| PE: ICD-9 code O14. \| \| E: ICD-9 code O15. \| |
| DeSisto 2021 | United States | \| GHTN: ICD-10 code O13. \| \| --- \| \| PE: ICD-10 code O14. \| \| E: ICD-10 code O15. \| |
| Passarella 2021 | United States | None provided. |
| Rozario 2021 | United States | PIH: the development of new hypertension during pregnancy after 20 weeks gestation, GHTN, PE, E, and unclassified HTN. |
| Xiao 2021 | United States | ICD-9-CM and ICD-10-CM codes. |
| Cameron 2022 | United States | None provided. |
| Chen 2022 | United States | ICD-9-CM or ICD-10-CM codes. |
| Feferkorn 2022 | United States | ICD-9-CM codes. |
| Felske 2022 | United States | None provided. |
| Ford 2022 | United States | \| GHTN: hypertension occurring after 20 weeks of gestation in persons with previously normal blood pressure \| \| --- \| \| Preeclampsia: gHTN with new-onset proteinuria. \| \| Eclampsia: new-onset tonic-clonic, focal, or multifocal seizures in the absence of other causative conditions \| \| cHTN with superimposed preeclampsia: preeclampsia in women with a history of hypertension before pregnancy or before 20 weeks of gestation. \| |
| Freaney 2022 | United States | \| HDP: both gestational hypertension and preeclampsia, did not include eclampsia or hemolysis, elevated liver enzymes, and low platelets syndrome. \| \| --- \| \| New-onset HDP is coded as yes or no and includes both gestational hypertension and preeclampsia together. \| |
| Gambahaya 2022 | United States | ICD-10-CM codes. |
| Guglielminotti 2022 | United States | ICD codes. |
| Huang 2022 | United States | ICD-9-CM and ICD-10-CM codes. |
| Kim 2022 | United States | ICD-9-CM and ICD-10-CM codes. |
| Kloppenburg 2022 | United States | \| Composite HDP: gestational hypertension, pre-eclampsia, and eclampsia. \| \| --- \| \| CHTN: defined as high blood pressure diagnosed prior to pregnancy or at less than 20 weeks of gestation. \| |
| Mazza 2022 | United States | Hypertensive disorders: gestational, pregestational, and preeclampsia. |
| McLaren 2022 | United States | Stage 1 hypertension: SBP> 130/80 mmHg. |
| Shen 2022 | United States | GHTN (including preeclampsia, transient hypertension of pregnancy, and pregnancy-induced hypertension). |
| Tarar 2022 | United States | ICD-10 codes |
| Thakkar 2022 | United States | ICD-9-CM and ICD-10-CM codes. |
| Wang 2022 | United States | ICD-9-CM and ICD-10-CM codes. |
| Wen 2022 | United States | ICD-9-CM and ICD-10-CM codes. |
| Wheeler 2022 | United States | ICD-9-CM and ICD-10-CM codes. |
| Wilson 2022 | United States | \| Preeclampsia: ICD-10 code O14. \| \| --- \| \| Eclampsia: ICD-10 code O15. \| \| GHTN: ICD-10 code O13. \| |
| Zahid 2022 | United States | ICD-9-CM and ICD-10-CM codes. |
| Azad 2023 | United States | ICD-9-CM and ICD-10-CM codes. |
| Christopher 2023 | United States | ICD-9 or ICD-10 codes. |
| Diab 2023 | United States | ICD-9-CM and ICD-10-CM codes. |
| Fink 2023 | United States | ICD-9-CM and ICD-10-CM codes. |
| Fronek 2023 | United States | HDP: either gestational hypertension or hypertension-eclampsia. |
| Goins 2023 | United States | ICD-9-CM and ICD-10-CM codes. |
| Green 2023 | United States | ICD-10-CM codes. |
| Huang 2023 | United States | ICD-9-CM and ICD-10-CM codes. |
| Kilani 2023 | United States | ICD-10-CM codes. |
| Lisonkova 2023 | United States | None provided. |
| Majmundar 2023 | United States | \| GHTN: ICD-10 code O13. \| \| --- \| \| Preeclampsia: ICD-10 code O14. \| \| Eclampsia: ICD-10 code O15. \| |
| Markson 2023 | United States | ICD-10-CM codes. |
| Mogos 2023 | United States | ICD-10-CM codes. |
| Niu 2023 | United States | HDP: defined as encompassing preeclampsia, eclampsia, and the hemolysis, elevated liver enzymes, and low platelets (HELLP) syndrome. |
| Niu 2023 | United States | Hypertensive complications: (preeclampsia, eclampsia, and HELLP). |
| Raina 2023 | United States | Hypertensive disorders of pregnancy (HDP, including gestational hypertension, preeclampsia, and eclampsia). |
| Sorokine 2023 | United States | ICD-9 codes. |
| Sun 2023 | United States | ICD-9-CM and ICD-10-CM codes. |
| Wang 2023 | United States | ICD-9-CM and ICD-10-CM codes. |
| Warner 2023 | United States | ICD-9-CM and ICD-10-CM codes. |
| Zahid 2023 | United States | ICD-9-CM and ICD-10-CM codes. |
| Briozzo 2023 | Uruguay | None provided. |

**Abbreviations:** WHO, World Health Organization; cHTN, chronic hypertension (before 20 weeks); GHTN, gestational hypertension (after 20 weeks); PE, preeclampsia; E, eclampsia; HDP, hypertensive disorders of pregnancy; HELLP, hemolysis, elevated liver enzymes, and low platelet count; ICD, International Classification of Diseases; SBP, systolic blood pressure; DBP, diastolic blood pressure

**Supplemental Table 3: Global Prevalence of Hypertensive Disorders of Pregnancy based on Population-based Studies by Condition and Study**

| **Study ID** | **Country** | **Chronic hypertension** | **Gestational hypertension** | **Preeclampsia** | **Eclampsia** | **Preeclampsia and Eclampsia** | **Gestational hypertension and Preeclampsia** | **Gestational hypertension, Preeclampsia and eclampsia** | **All Hypertensive Disorders of Pregnancy** |
| --- | --- | --- | --- | --- | --- | --- | --- | --- | --- |
| Wang 2016 | Australia |  |  |  |  |  | 4.38% |  |  |
| Kabir 2022 | Bangladesh |  |  |  |  |  |  |  | 24.80% |
| Souza 2020 | Brazil | 2.50% |  |  |  | 10.10% |  |  | 11.00% |
| Nakamura-Pereira 2023 | Brazil |  |  |  |  |  |  |  |  |
| Liu 2011 | Canada |  |  |  |  |  |  | 0.06% |  |
| Baghirzada 2022 | Canada |  |  | 6.50% | 0.10% |  |  |  |  |
| Sabr 2022 | Canada | 0.70% |  |  | 0.03% |  |  |  |  |
| Ye 2014 | China | 0.31% | 1.79% | 2.88% | 0.05% |  |  |  | 5.22% |
| Zhan 2021 | China |  |  |  |  |  |  |  | 5.60% |
| Wang 2022 | China |  |  |  |  |  |  |  | 4.10% |
| Sun 2023 | China |  |  |  |  |  |  |  | 13.10% |
| Wang 2023 | China |  | 1.50% |  |  | 1.40% |  |  |  |
| Vince 2021 | Croatia |  | 2.10% | 1.30% |  |  |  |  |  |
| Rode 2021 | Denmark | 0.50% |  | 3.20% |  |  |  |  |  |
| Aabakke 2023 | Denmark | 0.90% |  |  |  |  |  |  | 3.40% |
| Jaatinen 2016 | Finland |  |  | 1.50% | 0.02% |  |  |  |  |
| Bastola 2022 | Finland |  | 2.20% | 1.60% |  |  |  |  |  |
| Chen 2023 | Finland | 1.70% | 2.20% | 2.90% |  |  |  |  |  |
| Sesilia 2023 | Finland |  | 2.90% | 2% |  |  |  |  |  |
| Goueslard 2016 | France |  |  |  |  |  |  |  | 3.10% |
| Olie 2021 | France | 1.69%* | 4.2%* | 2.1%* | 0.07%* |  |  |  |  |
| Serrand 2021 | France |  |  |  |  | 1.10% |  |  |  |
| Martin 2022 | France | 4.70% |  |  |  |  |  | 5.90% |  |
| Blacher 2023 | France | 1.60% |  |  |  |  | 5.90% |  |  |
| Lailler 2023 | France | 1.50% |  | 2.90% |  |  |  |  |  |
| Lailler 2023 | France |  |  | 2.90% |  |  |  |  |  |
| Lailler 2023 | France | 1.54% | 3.86% | 2.41% |  |  |  |  | 7.81% |
| Lemaitre 2023 | France |  |  | 1.70% |  |  |  |  |  |
| Mettler 2023 | France |  |  |  |  |  |  |  | 4.80% |
| Schneider 2012 | Germany |  |  | 2.31% |  |  |  |  |  |
| Reinders 2020 | Germany | 6.10% | 3.30% | 6.10% |  |  |  |  |  |
| Weschenfelder 2023 | Germany |  |  |  |  |  |  |  |  |
| Papandreou 2022 | Greece |  |  |  |  |  |  |  |  |
| Papandreou 2023 | Greece |  |  |  |  |  |  |  |  |
| Pavlidou 2023 | Greece |  | 8.70% |  |  |  |  |  |  |
| Bridwell 2019 | Haiti | 1.20% | 3.23% | 1.35% |  |  |  | 5.80% |  |
| Eiríksdóttir · 2015 | Iceland |  | 2.90% | 3,3% |  |  |  |  | 5.90% |
| Singh 2018 | India |  |  |  |  |  |  |  | 3.67% |
| Chauhan 2023 | India |  |  |  |  |  |  |  |  |
| Grover 2023 | India |  |  |  |  |  |  |  |  |
| Corrigan 2021 | Ireland |  |  | 4.60% | 0.03% |  |  |  | 5.90% |
| Leitao 2022 | Ireland | 4.00% | 12.40% |  |  |  |  |  |  |
| Morikawa 2014 | Japan |  | 2.30% | 2.30% | 0.07% |  | 4.60% |  |  |
| Ishikawa 2023 | Japan |  |  |  |  |  |  |  |  |
| Khader 2018 | Jordan |  |  | 1.30% |  |  |  |  |  |
| Schaap 2019 | Netherlands |  |  |  | 0.18% |  |  |  |  |
| vanZijl 2020 | Netherlands |  |  |  |  |  |  |  | 10.50% |
| Rasmussen 2014 | Norway |  |  | 3.39% |  |  |  |  |  |
| Johnson 2016 | Botswana | 4.80% | 17.50% |  |  |  |  |  | 22.20% |
| Nurgaliyeva 2020 | Kazakhstan |  |  | 5.50% |  |  |  |  |  |
| Maducolil 2021 | Qatar | 1.10% | 1.70% | 2.39% |  |  |  |  | 5.10% |
| Al-Obaidly 2022 | Qatar |  |  | 1.60% |  |  |  |  |  |
| Lucovnik 2020 | Slovenia |  | 3.70% | 1.70% |  |  |  |  |  |
| Verschueren 2020 | Suriname |  |  |  | 0.78% |  |  |  |  |
| Sultan 2016 | Sweden |  |  |  |  | 3.63% |  |  |  |
| Liljestrom 2018 | Sweden |  |  |  |  |  |  |  | 4.30% |
| Mantel 2020 | Sweden |  |  | 2.70% |  |  |  |  |  |
| Byberg 2021 | Sweden |  |  |  |  | 2.80% |  |  |  |
| Yang 2021 | Sweden |  | 1.10% | 2.90% |  |  |  |  |  |
| Beer 2022 | Sweden | 0.70% |  | 2.70% |  |  |  |  |  |
| Byberg 2022 | Sweden |  |  | 2.80% |  |  |  |  |  |
| Dickmark 2022 | Sweden | 0.40% | 1.30% |  |  | 2.30% |  |  | 3.90% |
| Chang 2010 | Taiwan |  | 1.80% | 0.80% |  |  |  |  |  |
| Huang 2016 | Taiwan |  |  |  |  |  | 1.90% |  |  |
| Sun 2019 | Taiwan |  |  |  |  |  |  |  | 1.12% |
| Yu 2020 | Taiwan |  |  |  | 0.07% |  | 2.27% |  |  |
| Chen 2021 | Taiwan | 0.90% |  |  |  |  | 2.10% |  |  |
| Huang 2022 | Taiwan |  |  |  |  |  |  |  | 2.20% |
| Wu 2022 | Taiwan |  |  |  |  |  |  | 2.50% |  |
| Chen 2023 | Taiwan | 0.90% |  |  |  |  | 2.10% |  |  |
| Wang 2023 | Taiwan | 5.40% | 2.80% | 6.80% |  |  |  |  | 16.70% |
| Lim 2019 | South Korea |  |  |  |  | 1.25% |  |  |  |
| Noh 2020 | South Korea | 0.50% | 0.90% | 0.30% | 0.00% |  |  |  |  |
| Kim 2021 | South Korea |  | 1.90% |  |  |  |  |  |  |
| Cho 2022 | South Korea | 4.10% |  |  |  |  |  | 2.20% |  |
| Kim 2022 | South Korea |  |  | 0.20% |  |  |  |  |  |
| Kim 2022 | South Korea | 0.90% |  | 1.70% |  |  |  |  |  |
| Kim 2022 | South Korea | 5.40% | 3.10% |  |  | 1.80% |  |  | 9% |
| Kim 2022 | South Korea |  | 1.90%* |  |  |  |  |  |  |
| Kim 2022 | South Korea |  | 0.50% |  |  | 1.30%* |  |  |  |
| Park 2022 | South Korea |  |  |  |  |  |  | 1.90%* |  |
| Park 2022 | South Korea |  |  | 1.40% |  |  |  |  |  |
| Bae 2023 | South Korea |  |  |  |  |  |  |  | 2.70%* |
| Kim 2023 | South Korea |  |  | 1.60%* |  |  |  |  |  |
| Lee 2023 | South Korea |  |  |  |  |  |  |  |  |
| Lee 2023 | South Korea | 3.20% |  |  |  |  |  | 2% |  |
| Lee 2023 | South Korea |  | 1.30% |  |  |  |  |  |  |
| Lee 2023 | South Korea | 0.70%* |  |  |  |  |  |  |  |
| Nam 2023 | South Korea | 0.60% |  |  |  |  |  | 1.50% |  |
| Shim 2023 | South Korea | 0.70% |  | 2.50% |  |  |  |  |  |
| Lopez-de-Andres2020 | Spain |  | 1.10% |  |  |  |  |  |  |
| Knight 2007 | United Kingdom |  |  | 2.70%* |  |  |  |  |  |
| Kayem 2011 | United Kingdom |  |  |  | 0.02% |  |  |  |  |
| Khandwala 2018 | United States |  |  | 4.60% | 0.20% |  |  |  |  |
| Rossi 2019 | United States |  |  |  |  |  | 5.30% |  |  |
| Tripathi 2019 | United States |  |  |  |  |  |  | 9.71% |  |
| DeSisto 2021 | United States | 2.2 | 4.7 |  |  | 4.8 |  |  |  |
| Passarella 2021 | United States |  |  | 4.20% | 0.10% |  |  |  |  |
| Rozario 2021 | United States |  | 5% |  |  |  |  |  |  |
| Xiao 2021 | United States | 1.51% |  |  | 0.30% |  |  |  | 5.04% |
| Cameron 2022 | United States |  |  |  |  |  |  | 8.30%* |  |
| Chen 2022 | United States |  |  |  |  | 5.30% |  |  |  |
| Feferkorn 2022 | United States | 1.80% | 3.30% |  |  | 4.20% |  |  |  |
| Felske 2022 | United States | 2.20% | 7.70% |  | 0.30% |  |  |  |  |
| Ford 2022 | United States | 2.30% |  |  |  |  |  | 13.00% | 15.90% |
| Freaney 2022 | United States |  |  |  |  |  | 7.78%* |  |  |
| Gambahaya 2022 | United States | 0.30% | 5.40% |  |  | 5.30% |  |  |  |
| Guglielminotti 2022 | United States | 1.90% | 6.50% |  | 0.28%* |  |  |  |  |
| Huang 2022 | United States | 3.10% | 3.90% |  |  |  |  |  |  |
| Kim 2022 | United States | 1.60% |  |  | 0.20% |  |  |  |  |
| Kloppenburg 2022 | United States | 1.90%* |  |  | 0.25%* |  | 6.42%* |  |  |
| Mazza 2022 | United States | 3.60% | 5.20% | 5.20% |  |  |  |  |  |
| McLaren 2022 | United States | 2.20% |  |  |  |  |  |  |  |
| Shen 2022 | United States |  |  |  |  |  | 10% |  |  |
| Tarar 2022 | United States |  | 2.10% |  |  |  |  |  |  |
| Thakkar 2022 | United States | 1.50% |  | 3.60% |  |  |  |  |  |
| Wang 2022 | United States |  | 6.60% |  | 0.20% |  |  |  |  |
| Wen 2022 | United States |  |  |  |  |  |  | 12% |  |
| Wheeler 2022 | United States |  |  |  |  |  |  |  |  |
| Wilson 2022 | United States | 4.10% | 3.50% | 3.60% | 0.10% |  |  |  |  |
| Zahid 2022 | United States | 0.70% |  |  |  | 4.50% |  |  |  |
| Azad 2023 | United States | 2.00% | 2.60% | 4.90% |  |  |  |  |  |
| Christopher 2023 | United States | 2.80% | 4.40% |  |  | 5.10% |  |  |  |
| Diab 2023 | United States | 2.80% | 9.50% |  | 0.30% |  |  |  |  |
| Fink 2023 | United States | 2.70% | 5.00% | 5.80% | 0.10% |  |  |  |  |
| Fronek 2023 | United States | 1% |  |  |  |  |  |  |  |
| Goins 2023 | United States | 3.50% |  |  |  |  | 12.20% |  |  |
| Green 2023 | United States |  |  |  | 0.14%* |  |  |  |  |
| Huang 2023 | United States | 3.90% |  |  |  |  | 13.20% |  |  |
| Kilani 2023 | United States |  |  |  |  |  |  |  |  |
| Lisonkova 2023 | United States | 2.70% |  |  |  |  |  |  |  |
| Majmundar 2023 | United States |  | 5.46% | 5.46% | 0.11% |  |  | 10.69% |  |
| Markson 2023 | United States |  |  | 1.80% |  |  |  |  |  |
| Mogos 2023 | United States |  |  |  |  |  |  |  |  |
| Niu 2023 | United States | 2.90% |  |  |  |  |  |  | 5.10% |
| Niu 2023 | United States |  |  |  |  |  |  |  | 0.06% |
| Raina 2023 | United States | 1.80% | 3.30% | 3.60% |  |  |  | 6.90% |  |
| Sorokine 2023 | United States |  |  | 4.20% | 0.10% |  |  |  |  |
| Sun 2023 | United States |  | 5.60% |  | 0.20% |  |  |  |  |
| Wang 2023 | United States | 2.10% | 7.80% |  | 0.30% |  |  |  |  |
| Warner 2023 | United States |  |  | 6.60% | 0.10% |  |  |  |  |
| Zahid 2023 | United States | 0.50% |  |  |  | 4.90% |  |  |  |
| Briozzo 2023 | Uruguay | 2.50% | 8.00% |  |  | 4.90% |  |  |  |

*Age-adjusted prevalence estimates.

**Supplemental Table 4. Global Prevalence of Hypertensive Disorders of Pregnancy by Country/Jurisdictions**

| **WHO. Region** | **Country** | **Chronic hypertension** | **Gestational hypertension** | **Preeclampsia** | **Eclampsia** | **Preeclampsia and Eclampsia** | **Gestational hypertension and Preeclampsia** | **Gestational hypertension, Preeclampsia and eclampsia** | **All Hypertensive Disorders of Pregnancy** |
| --- | --- | --- | --- | --- | --- | --- | --- | --- | --- |
| **African Region** | Botswana | 4.80% | 17.50% |  |  |  |  |  | 22.20% |
| **Americas Region** | Brazil | 2.50% |  |  |  | 10.10% |  |  | 11.00% |
|  | Canada | 0.70% |  | 6.50% | 0.03% |  |  | 0.06% |  |
|  | Haiti | 1.20% | 3.23% | 1.35% |  |  |  | 5.80% |  |
|  | Suriname |  |  |  |  |  | 0.78% |  |  |
|  | United States | 2.35% | 5.10% | 5.35% | 0.13% | 4.50% | 10%* | 10.69%* | 2.55% |
|  | Uruguay | 2.50% | 8.00% |  |  | 4.90% |  |  |  |
| **Eastern Mediterranean Region** | Jordan |  |  | 1.30% |  |  |  |  | 5.10% |
|  | Qatar | 1.10% | 1.70% | 1.60% |  |  |  |  |  |
| **European Region** | Croatia |  | 2.10% | 1.30% |  |  |  |  |  |
|  | Denmark | 0.70% | 2.10% | 2.25% |  |  |  |  | 3.40% |
|  | Finland | 1.70% | 2.20% | 2.00% | 0.02% |  |  |  |  |
|  | France | 1.70%* | 4.05%* | 2%* | 0.07%* | 1.10% | 5.90% | 5.90% | 5.50% |
|  | Germany | 6.10% | 3.30% | 4.21% |  |  |  |  |  |
|  | Greece |  | 8.70% |  |  |  |  |  |  |
|  | Iceland |  | 2.90% | 3.30% |  |  |  |  | 5.90% |
|  | Ireland | 4% | 12.40% | 4.60% | 0.03% |  |  |  | 5.90% |
|  | Kazakhstan |  |  |  |  | 5.50% |  |  |  |
|  | Netherlands |  |  |  | 0.02% |  |  |  | 10.50% |
|  | Norway |  |  |  |  | 3.39% |  |  |  |
|  | Slovenia |  | 3.70% | 1.70% |  |  |  |  |  |
|  | Spain |  | 1.10% |  |  |  |  |  |  |
|  | Sweden | 0.55% | 1.20% | 2.75% |  | 2.80% |  |  | 4.30% |
|  | United Kingdom |  |  | 0.27% | 0.02% |  |  |  |  |
| **South-East Asian Region** | Bangladesh |  |  |  |  |  |  |  | 24.80% |
|  | India |  |  |  |  |  |  |  | 3.67% |
| **Western Pacific Region** | Australia |  |  |  |  |  | 4.38% |  |  |
|  | China | 0.31% | 1.65% | 2.59% | 0.05% | 1.40% |  |  | 5.60% |
|  | Japan |  | 2.30% | 2.30% | 0.07% |  | 4.60% |  |  |
|  | South Korea | 4.10% | 1.30% | 1.60% | 0.00% | 1.53% |  | 2.05% | 5.85% |
|  | Taiwan | 3.15% | 2.30% | 3.80% | 0.07% |  | 2.10% | 2.50% | 2.20% |

*Age-adjusted prevalence estimates.

**Supplemental Table 5. Global Prevalence of Hypertensive Disorders of Pregnancy by World Health Organization Region**

| **WHO. Region** | **Chronic hypertension** | **Gestational hypertension** | **Preeclampsia** | **Eclampsia** | **Preeclampsia and Eclampsia** | **Gestational hypertension and Preeclampsia** | **Gestational hypertension, Preeclampsia and eclampsia** | **All Hypertensive Disorders of Pregnancy** |
| --- | --- | --- | --- | --- | --- | --- | --- | --- |
| **African Region** | 4.80% | 17.50% | NA | NA | NA | NA | NA | 22.20% |
| **Americas Region** | 2.35% | 5.10% | 5.35% | 0.13% | 4.90% | 10.00% | 5.80% | 6.78% |
| **Eastern Mediterranean Region** | 1.10% | 1.70% | 1.45% | NA | NA | NA | NA | 5.10% |
| **European Region** | 1.70% | 2.90% | 2.50% | 0.02% | 1.95% | 5.90% | 5.90% | 5.90% |
| **South-East Asian Region** | NA | NA | NA | NA | NA | NA | NA | 14.24% |
| **Western Pacific Region** | 3.15% | 1.98% | 2.45% | 0.06% | 1.45% | 4.38% | 2.28% | 5.60% |

**Supplemental Table 6: Quality Assessment of All Papers Included in the Review**

| **Study ID** | **Country** | **How was the  cohort selected?** | **Is the study sample size  (final analytic n) >= 2500?** | **How is HDP  assessed?** | **Did the study report  age-standardized  HDP prevalence?** | **How was prevalence  of HDP reported?** | **Aggregate score** | **Final quality score** |
| --- | --- | --- | --- | --- | --- | --- | --- | --- |
| Wang 2016 | Australia | 1 | 1 | 0 | 0 | 0 | 2 | Fair |
| Kabir 2022 | Bangladesh | 1 | 0 | 0 | 0 | 0 | 1 | Poor |
| Souza 2020 | Brazil | 1 | 1 | 1 | 0 | 1 | 4 | Good |
| Nakamura-Pereira 2023 | Brazil | 1 | 1 | 1 | 0 | 0 | 3 | Fair |
| Liu 2011 | Canada | 1 | 1 | 1 | 0 | 0 | 3 | Fair |
| Baghirzada 2022 | Canada | 1 | 1 | 1 | 0 | 1 | 4 | Good |
| Sabr 2022 | Canada | 1 | 1 | 1 | 0 | 1 | 4 | Good |
| Ye 2014 | China | 1 | 1 | 1 | 0 | 1 | 4 | Good |
| Zhan 2021 | China | 1 | 1 | 1 | 0 | 0 | 3 | Fair |
| Wang 2022 | China | 1 | 1 | 1 | 0 | 0 | 3 | Fair |
| Sun 2023 | China | 0 | 1 | 0 | 0 | 0 | 1 | Poor |
| Wang 2023 | China | 1 | 1 | 1 | 0 | 1 | 4 | Good |
| Vince 2021 | Croatia | 1 | 1 | 1 | 0 | 1 | 4 | Good |
| Rode 2021 | Denmark | 1 | 1 | 1 | 0 | 0 | 3 | Fair |
| Aabakke 2023 | Denmark | 1 | 1 | 1 | 0 | 0 | 3 | Fair |
| Jaatinen 2016 | Finland | 1 | 1 | 1 | 0 | 0 | 3 | Fair |
| Bastola 2022 | Finland | 1 | 1 | 0 | 0 | 1 | 3 | Fair |
| Chen 2023 | Finland | 1 | 1 | 1 | 0 | 1 | 4 | Good |
| Sesilia 2023 | Finland | 1 | 1 | 1 | 0 | 1 | 4 | Good |
| Goueslard 2016 | France | 1 | 1 | 1 | 0 | 0 | 3 | Fair |
| Olie 2021 | France | 1 | 1 | 1 | 0 | 1 | 4 | Good |
| Serrand 2021 | France | 1 | 1 | 1 | 0 | 0 | 3 | Fair |
| Martin 2022 | France | 1 | 1 | 1 | 0 | 1 | 4 | Good |
| Blacher 2023 | France | 1 | 1 | 1 | 0 | 1 | 4 | Good |
| Lailler 2023 | France | 1 | 1 | 1 | 0 | 1 | 4 | Good |
| Lailler 2023 | France | 1 | 1 | 1 | 0 | 0 | 3 | Fair |
| Lailler 2023 | France | 1 | 1 | 1 | 0 | 1 | 4 | Good |
| Lemaitre 2023 | France | 1 | 1 | 1 | 0 | 0 | 3 | Fair |
| Mettler 2023 | France | 1 | 1 | 1 | 0 | 0 | 3 | Fair |
| Schneider 2012 | Germany | 1 | 1 | 1 | 0 | 0 | 3 | Fair |
| Reinders 2020 | Germany | 1 | 1 | 1 | 0 | 1 | 4 | Good |
| Weschenfelder 2023 | Germany | 0 | 0 | 1 | 0 | 0 | 1 | Poor |
| Papandreou 2022 | Greece | 1 | 1 | 1 | 0 | 0 | 3 | Fair |
| Papandreou 2023 | Greece | 1 | 1 | 1 | 0 | 0 | 3 | Fair |
| Pavlidou 2023 | Greece | 1 | 1 | 1 | 0 | 0 | 3 | Fair |
| Bridwell 2019 | Haiti | 1 | 1 | 1 | 0 | 1 | 4 | Good |
| Eiríksdóttir · 2015 | Iceland | 1 | 1 | 1 | 0 | 1 | 4 | Good |
| Singh 2018 | India | 1 | 1 | 1 | 0 | 0 | 3 | Fair |
| Chauhan 2023 | India | 1 | 1 | 0 | 0 | 0 | 2 | Fair |
| Grover 2023 | India | 1 | 1 | 0 | 0 | 0 | 2 | Fair |
| Corrigan 2021 | Ireland | 1 | 1 | 1 | 0 | 1 | 4 | Good |
| Leitao 2022 | Ireland | 1 | 1 | 1 | 0 | 0 | 3 | Fair |
| Morikawa 2014 | Japan | 1 | 1 | 1 | 0 | 1 | 4 | Good |
| Ishikawa 2023 | Japan | 1 | 1 | 1 | 0 | 0 | 3 | Fair |
| Khader 2018 | Jordan | 1 | 1 | 1 | 0 | 0 | 3 | Fair |
| Schaap 2019 | Netherlands | 1 | 1 | 1 | 0 | 0 | 3 | Fair |
| VanZijl 2020 | Netherlands | 1 | 1 | 1 | 0 | 0 | 3 | Fair |
| Rasmussen 2014 | Norway | 1 | 1 | 1 | 0 | 0 | 3 | Fair |
| Johnson 2016 | Botswana | 1 | 1 | 1 | 0 | 1 | 4 | Good |
| Nurgaliyeva 2020 | Kazakhstan | 1 | 1 | 1 | 0 | 0 | 3 | Fair |
| Maducolil 2021 | Qatar | 1 | 1 | 1 | 0 | 1 | 4 | Good |
| Al-Obaidly 2022 | Qatar | 1 | 1 | 1 | 0 | 0 | 3 | Fair |
| Lucovnik 2020 | Slovenia | 1 | 1 | 1 | 0 | 1 | 4 | Good |
| Verschueren 2020 | Suriname | 1 | 1 | 1 | 0 | 0 | 3 | Fair |
| Sultan 2016 | Sweden | 1 | 1 | 1 | 0 | 0 | 3 | Fair |
| Liljestrom 2018 | Sweden | 1 | 1 | 1 | 0 | 0 | 3 | Fair |
| Mantel 2020 | Sweden | 1 | 1 | 1 | 0 | 0 | 3 | Fair |
| Byberg 2021 | Sweden | 1 | 1 | 1 | 0 | 0 | 3 | Fair |
| Yang 2021 | Sweden | 1 | 1 | 1 | 0 | 0 | 3 | Fair |
| Beer 2022 | Sweden | 1 | 1 | 1 | 0 | 1 | 4 | Good |
| Byberg 2022 | Sweden | 1 | 1 | 1 | 0 | 0 | 3 | Fair |
| Dickmark 2022 | Sweden | 1 | 1 | 1 | 0 | 1 | 4 | Good |
| Chang 2010 | Taiwan | 1 | 1 | 1 | 0 | 1 | 4 | Good |
| Huang 2016 | Taiwan | 1 | 1 | 1 | 0 | 0 | 3 | Fair |
| Sun 2019 | Taiwan | 1 | 1 | 1 | 0 | 0 | 3 | Fair |
| Yu 2020 | Taiwan | 1 | 1 | 1 | 0 | 1 | 4 | Good |
| Chen 2021 | Taiwan | 1 | 1 | 1 | 0 | 1 | 4 | Good |
| Huang 2022 | Taiwan | 1 | 1 | 1 | 0 | 0 | 3 | Fair |
| Wu 2022 | Taiwan | 1 | 1 | 1 | 0 | 0 | 3 | Fair |
| Chen 2023 | Taiwan | 1 | 1 | 1 | 0 | 1 | 4 | Good |
| Wang 2023 | Taiwan | 1 | 1 | 1 | 0 | 1 | 4 | Good |
| Lim 2019 | South Korea | 1 | 1 | 1 | 0 | 0 | 3 | Fair |
| Noh 2020 | South Korea | 1 | 1 | 1 | 0 | 1 | 4 | Good |
| Kim 2021 | South Korea | 1 | 1 | 1 | 0 | 0 | 3 | Fair |
| Cho 2022 | South Korea | 1 | 1 | 1 | 0 | 0 | 3 | Fair |
| Kim 2022 | South Korea | 1 | 1 | 1 | 0 | 0 | 3 | Fair |
| Kim 2022 | South Korea | 1 | 1 | 1 | 0 | 1 | 4 | Good |
| Kim 2022 | South Korea | 1 | 1 | 1 | 1 | 1 | 5 | Good |
| Kim 2022 | South Korea | 0 | 1 | 1 | 0 | 0 | 2 | Fair |
| Kim 2022 | South Korea | 1 | 1 | 1 | 0 | 1 | 4 | Good |
| Park 2022 | South Korea | 1 | 1 | 1 | 0 | 0 | 3 | Fair |
| Park 2022 | South Korea | 1 | 1 | 1 | 0 | 0 | 3 | Fair |
| Bae 2023 | South Korea | 1 | 1 | 1 | 0 | 0 | 3 | Fair |
| Kim 2023 | South Korea | 1 | 1 | 1 | 0 | 0 | 3 | Fair |
| Lee 2023 | South Korea | 1 | 1 | 1 | 0 | 0 | 3 | Fair |
| Lee 2023 | South Korea | 1 | 1 | 1 | 0 | 0 | 3 | Fair |
| Lee 2023 | South Korea | 1 | 1 | 1 | 0 | 0 | 3 | Fair |
| Lee 2023 | South Korea | 1 | 1 | 1 | 0 | 0 | 3 | Fair |
| Nam 2023 | South Korea | 1 | 1 | 1 | 0 | 0 | 3 | Fair |
| Shim 2023 | South Korea | 1 | 1 | 1 | 0 | 1 | 4 | Good |
| Lopez-de-Andres 2020 | Spain | 1 | 1 | 1 | 0 | 0 | 3 | Fair |
| Knight 2007 | United Kingdom | 1 | 0 | 0 | 0 | 0 | 1 | Poor |
| Kayem 2011 | United Kingdom | 1 | 1 | 1 | 0 | 0 | 3 | Fair |
| Khandwala 2018 | USA | 1 | 1 | 1 | 0 | 1 | 4 | Good |
| Rossi 2019 | USA | 1 | 1 | 1 | 0 | 0 | 3 | Fair |
| Tripathi 2019 | USA | 1 | 1 | 1 | 0 | 0 | 3 | Fair |
| DeSisto 2021 | USA | 1 | 1 | 1 | 0 | 1 | 4 | Good |
| Passarella 2021 | USA | 1 | 1 | 1 | 0 | 1 | 4 | Good |
| Rozario 2021 | USA | 1 | 1 | 1 | 0 | 0 | 3 | Fair |
| Xiao 2021 | USA | 1 | 1 | 1 | 0 | 1 | 4 | Good |
| Cameron 2022 | USA | 1 | 1 | 1 | 1 | 0 | 4 | Good |
| Chen 2022 | USA | 1 | 1 | 1 | 0 | 0 | 3 | Fair |
| Feferkorn 2022 | USA | 1 | 1 | 1 | 0 | 1 | 4 | Good |
| Felske 2022 | USA | 1 | 1 | 1 | 0 | 1 | 4 | Good |
| Ford 2022 | USA | 1 | 1 | 1 | 0 | 1 | 4 | Good |
| Freaney 2022 | USA | 1 | 1 | 1 | 1 | 0 | 4 | Good |
| Gambahaya 2022 | USA | 1 | 1 | 1 | 0 | 1 | 4 | Good |
| Guglielminotti 2022 | USA | 1 | 1 | 1 | 0 | 1 | 4 | Good |
| Huang 2022 | USA | 1 | 1 | 1 | 0 | 1 | 4 | Good |
| Kim 2022 | USA | 1 | 1 | 1 | 0 | 1 | 4 | Good |
| Kloppenburg 2022 | USA | 1 | 1 | 1 | 0 | 1 | 4 | Good |
| Mazza 2022 | USA | 1 | 1 | 1 | 0 | 1 | 4 | Good |
| McLaren 2022 | USA | 1 | 1 | 1 | 0 | 0 | 3 | Fair |
| Shen 2022 | USA | 1 | 1 | 1 | 0 | 0 | 3 | Fair |
| Tarar 2022 | USA | 1 | 1 | 1 | 0 | 0 | 3 | Fair |
| Thakkar 2022 | USA | 1 | 1 | 1 | 0 | 1 | 4 | Good |
| Wang 2022 | USA | 1 | 1 | 1 | 0 | 1 | 4 | Good |
| Wen 2022 | USA | 1 | 1 | 1 | 0 | 0 | 3 | Fair |
| Wheeler 2022 | USA | 1 | 1 | 1 | 0 | 0 | 3 | Fair |
| Wilson 2022 | USA | 1 | 1 | 1 | 0 | 1 | 4 | Good |
| Zahid 2022 | USA | 1 | 1 | 1 | 0 | 1 | 4 | Good |
| Azad 2023 | USA | 1 | 1 | 1 | 0 | 1 | 4 | Good |
| Christopher 2023 | USA | 1 | 1 | 1 | 0 | 1 | 4 | Good |
| Diab 2023 | USA | 1 | 1 | 1 | 0 | 1 | 4 | Good |
| Fink 2023 | USA | 1 | 1 | 1 | 0 | 1 | 4 | Good |
| Fronek 2023 | USA | 1 | 1 | 1 | 0 | 0 | 3 | Fair |
| Goins 2023 | USA | 1 | 1 | 1 | 0 | 1 | 4 | Good |
| Green 2023 | USA | 1 | 1 | 1 | 0 | 0 | 3 | Fair |
| Huang 2023 | USA | 1 | 1 | 1 | 0 | 1 | 4 | Good |
| Kilani 2023 | USA | 1 | 1 | 1 | 0 | 0 | 3 | Fair |
| Lisonkova 2023 | USA | 1 | 1 | 1 | 0 | 0 | 3 | Fair |
| Majmundar 2023 | USA | 1 | 1 | 1 | 0 | 0 | 3 | Fair |
| Markson 2023 | USA | 1 | 1 | 1 | 0 | 1 | 4 | Good |
| Mogos 2023 | USA | 1 | 1 | 1 | 0 | 0 | 3 | Fair |
| Niu 2023 | USA | 1 | 1 | 1 | 0 | 1 | 4 | Good |
| Niu 2023 | USA | 1 | 1 | 1 | 0 | 0 | 3 | Fair |
| Raina 2023 | USA | 1 | 1 | 1 | 0 | 1 | 4 | Good |
| Sorokine 2023 | USA | 1 | 1 | 1 | 0 | 1 | 4 | Good |
| Sun 2023 | USA | 1 | 1 | 1 | 0 | 1 | 4 | Good |
| Wang 2023 | USA | 1 | 1 | 1 | 0 | 1 | 4 | Good |
| Warner 2023 | USA | 1 | 1 | 1 | 0 | 1 | 4 | Good |
| Zahid 2023 | USA | 1 | 1 | 1 | 0 | 1 | 4 | Good |
| Briozzo 2023 | Uruguay | 1 | 1 | 1 | 0 | 1 | 4 | Good |

**Abbreviation:** HDP, hypertensive disorders of pregnancy

**Supplemental Table 7. Income Group Classification by Country/Jurisdictions Included in this Review Paper [19]**

| **World Health Organization Region** | **Country** | **Income Group** |
| --- | --- | --- |
| **African Region** | Botswana | Upper middle income |
| **Americas Region** | United States | High income |
|  | Brazil | Upper middle income |
|  | Canada | High income |
|  | Haiti | Lower middle income |
|  | Suriname | Upper middle income |
|  | Uruguay | High income |
| **Eastern Mediterranean Region** | Qatar | High income |
|  | Jordan | Lower middle income |
| **European Region** | France | High income |
|  | Sweden | High income |
|  | Finland | High income |
|  | Germany | High income |
|  | Greece | High income |
|  | Denmark | High income |
|  | Ireland | High income |
|  | Netherlands | High income |
|  | United Kingdom | High income |
|  | Croatia | High income |
|  | Iceland | High income |
|  | Norway | High income |
|  | Kazakhstan | Upper middle income |
|  | Slovenia | High income |
|  | Spain | High income |
| **South-East Asian Region** | India | Lower middle income |
|  | Bangladesh | Lower middle income |
| **Western Pacific Region** | South Korea | High income |
|  | Taiwan | High income |
|  | China | Upper middle income |
|  | Japan | High income |
|  | Australia | High income |

**Supplemental Figure 1: Global Prevalence of (A) Chronic Hypertension, (B) Eclampsia, (C) Preeclampsia and Eclampsia, (D) Gestational Hypertension and Preeclampsia, (E) Gestational Hypertension,** **Preeclampsia, and Eclampsia by Country/Jurisdictions**

1. **Chronic Hypertension Before Pregnancy or During Early Pregnancy**

**Supplemental Figure 1: Global Prevalence of (A) Chronic Hypertension, (B) Eclampsia, (C) Preeclampsia and Eclampsia, (D) Gestational Hypertension and Preeclampsia, (E) Gestational Hypertension,** **Preeclampsia, and Eclampsia by Country/Jurisdictions**

1. **Eclampsia**

**Supplemental Figure 1: Global Prevalence of (A) Chronic Hypertension, (B) Eclampsia, (C) Preeclampsia and Eclampsia, (D) Gestational Hypertension and Preeclampsia, (E) Gestational Hypertension,** **Preeclampsia, and Eclampsia by Country/Jurisdictions**

1. **Preeclampsia and Eclampsia**

**Supplemental Figure 1: Global Prevalence of (A) Chronic Hypertension, (B) Eclampsia, (C) Preeclampsia and Eclampsia, (D) Gestational Hypertension and Preeclampsia, (E) Gestational Hypertension,** **Preeclampsia, and Eclampsia by Country/Jurisdictions**

1. **Gestational Hypertension and Preeclampsia**

**Supplemental Figure 1: Global Prevalence of (A) Chronic Hypertension, (B) Eclampsia, (C) Preeclampsia and Eclampsia, (D) Gestational Hypertension and Preeclampsia, (E) Gestational Hypertension,** **Preeclampsia, and Eclampsia by Country/Jurisdictions**

1. **Gestational Hypertension, Preeclampsia, and Eclampsia**

**Supplemental Figure 2: Global Prevalence of (A) Chronic Hypertension, (B) Eclampsia, (C) Preeclampsia and Eclampsia, (D) Gestational Hypertension and Preeclampsia, (E) Gestational Hypertension,** **Preeclampsia, and Eclampsia by World Health Organization Region**

1. **Chronic Hypertension Before Pregnancy or During Early Pregnancy**


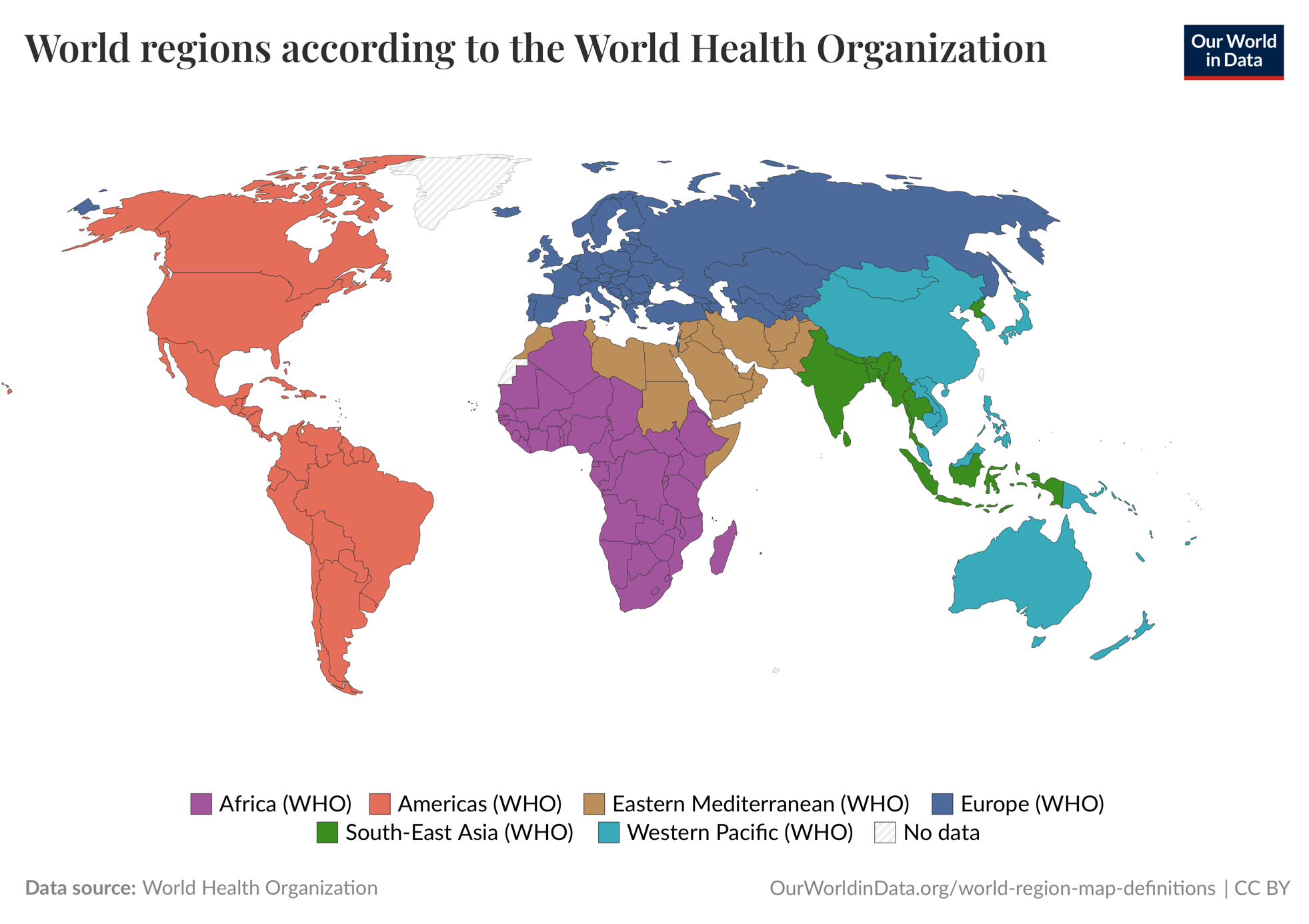


**Western Pacific
3.15%**

**Europe
1.70%**

**South-East Asia
No data**

**Eastern Mediterranean
1.10%**

**Africa
4.80%**

**Americas
2.35%**

**Supplemental Figure 2: Global Prevalence of (A) Chronic Hypertension, (B) Eclampsia, (C) Preeclampsia and Eclampsia, (D) Gestational Hypertension and Preeclampsia, (E) Gestational Hypertension,** **Preeclampsia, and Eclampsia by World Health Organization Region**

1. **Eclampsia**


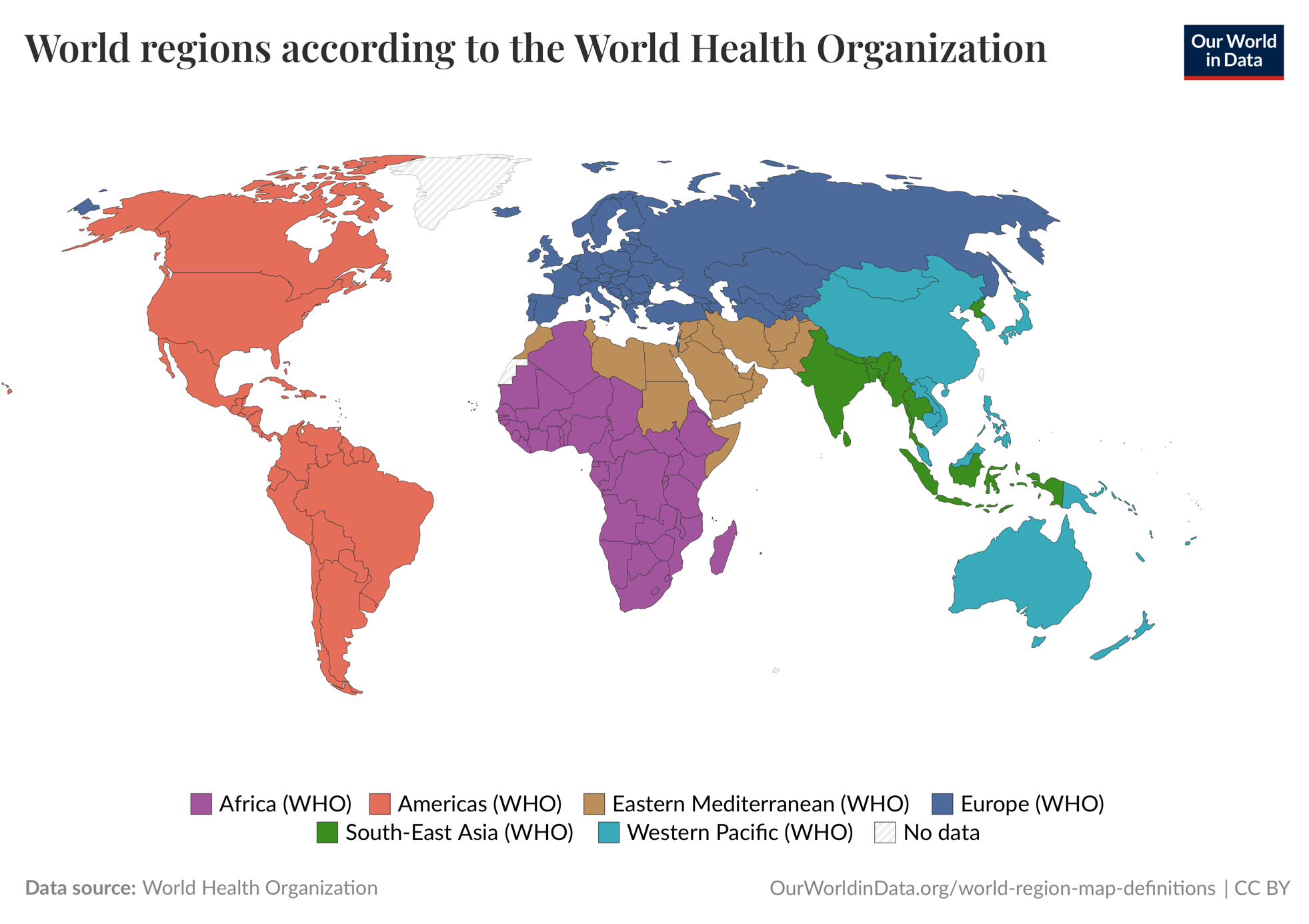


**Western Pacific
0.06%**

**Europe
0.02%**

**South-East Asia
No data**

**Eastern Mediterranean
No data**

**Africa
No data**

**Americas
0.13%**

**Supplemental Figure 2: Global Prevalence of (A) Chronic Hypertension, (B) Eclampsia, (C) Preeclampsia and Eclampsia, (D) Gestational Hypertension and Preeclampsia, (E) Gestational Hypertension,** **Preeclampsia, and Eclampsia by World Health Organization Region**

1. **Preeclampsia and Eclampsia**

**South-East Asia
No data**


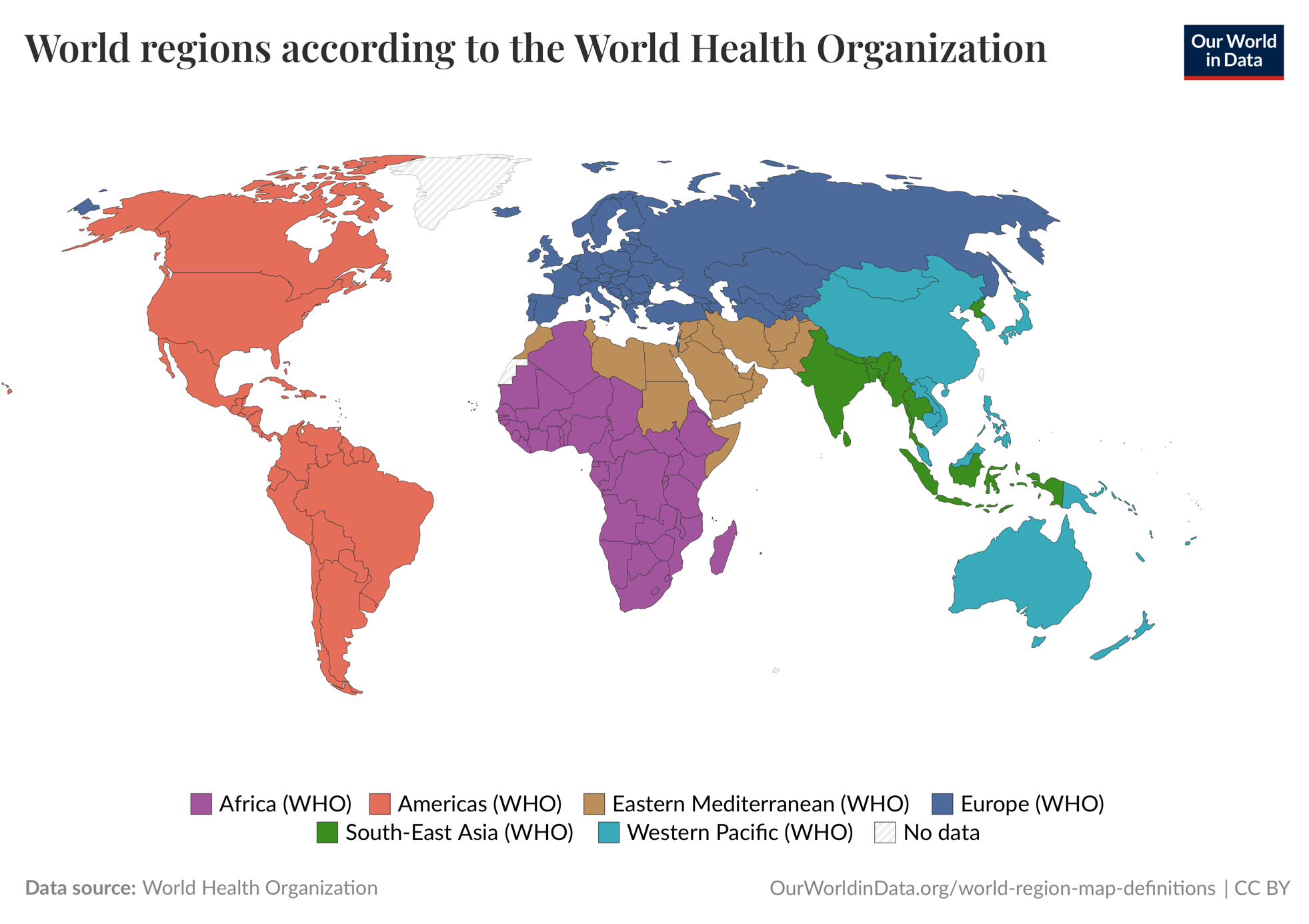


**Western Pacific
1.45%**

**Europe
1.95%**

**Americas
4.90%**

**Eastern Mediterranean
No data**

**Africa
No data**

**Supplemental Figure 2: Global Prevalence of (A) Chronic Hypertension, (B) Eclampsia, (C) Preeclampsia and Eclampsia, (D) Gestational Hypertension and Preeclampsia, (E) Gestational Hypertension,** **Preeclampsia, and Eclampsia by World Health Organization Region**

1. **Gestational Hypertension and Preeclampsia**

**South-East Asia
No data**

**Eastern Mediterranean
No data**

**Africa
No data**


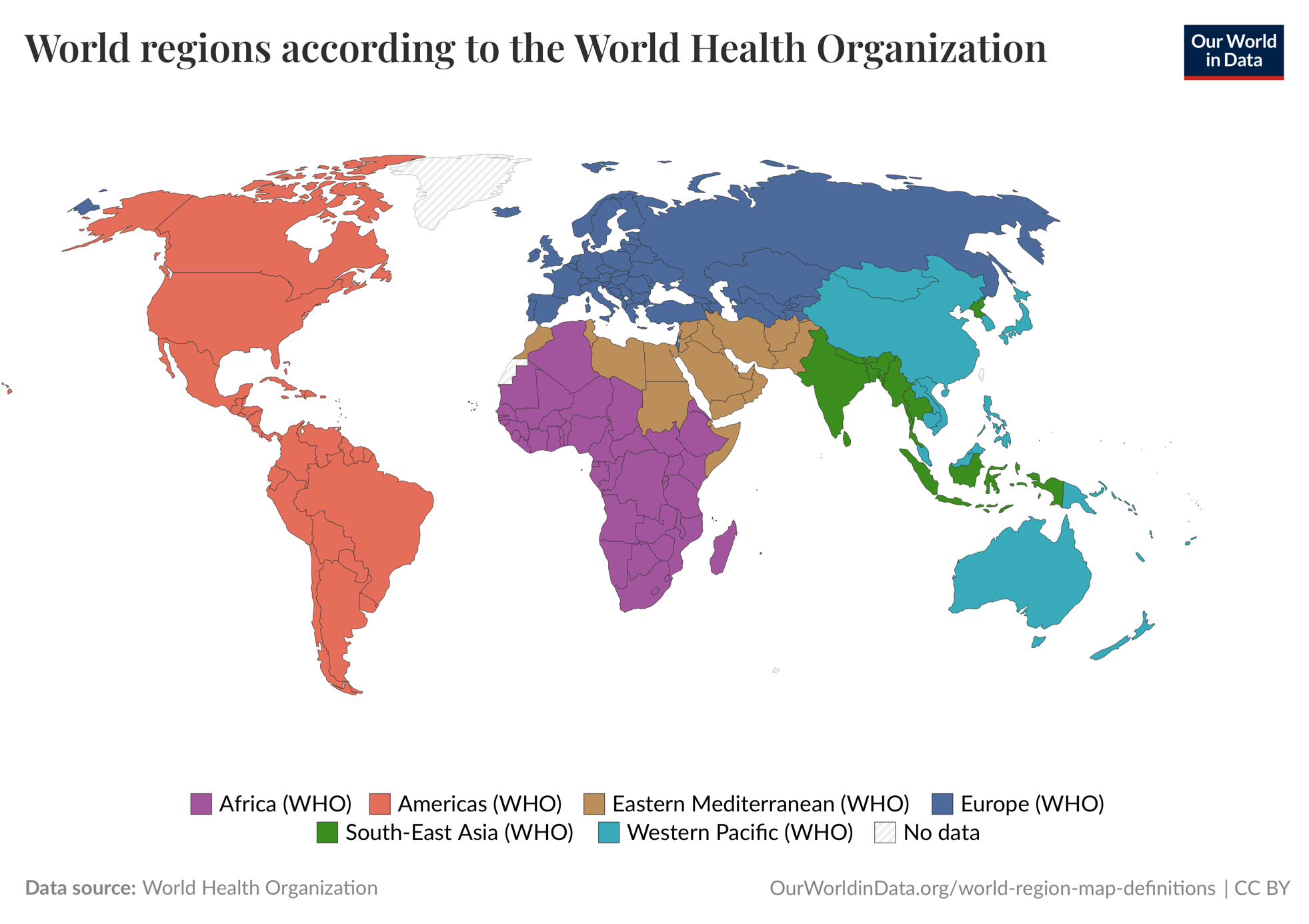


**Western Pacific
4.38%**

**Europe
5.90%**

**Americas
10.00%**

**Supplemental Figure 2: Global Prevalence of (A) Chronic Hypertension, (B) Eclampsia, (C) Preeclampsia and Eclampsia, (D) Gestational Hypertension and Preeclampsia, (E) Gestational Hypertension,** **Preeclampsia, and Eclampsia by World Health Organization Region**

**Africa
No data**

**Eastern Mediterranean
No data**

**South-East Asia
No data**


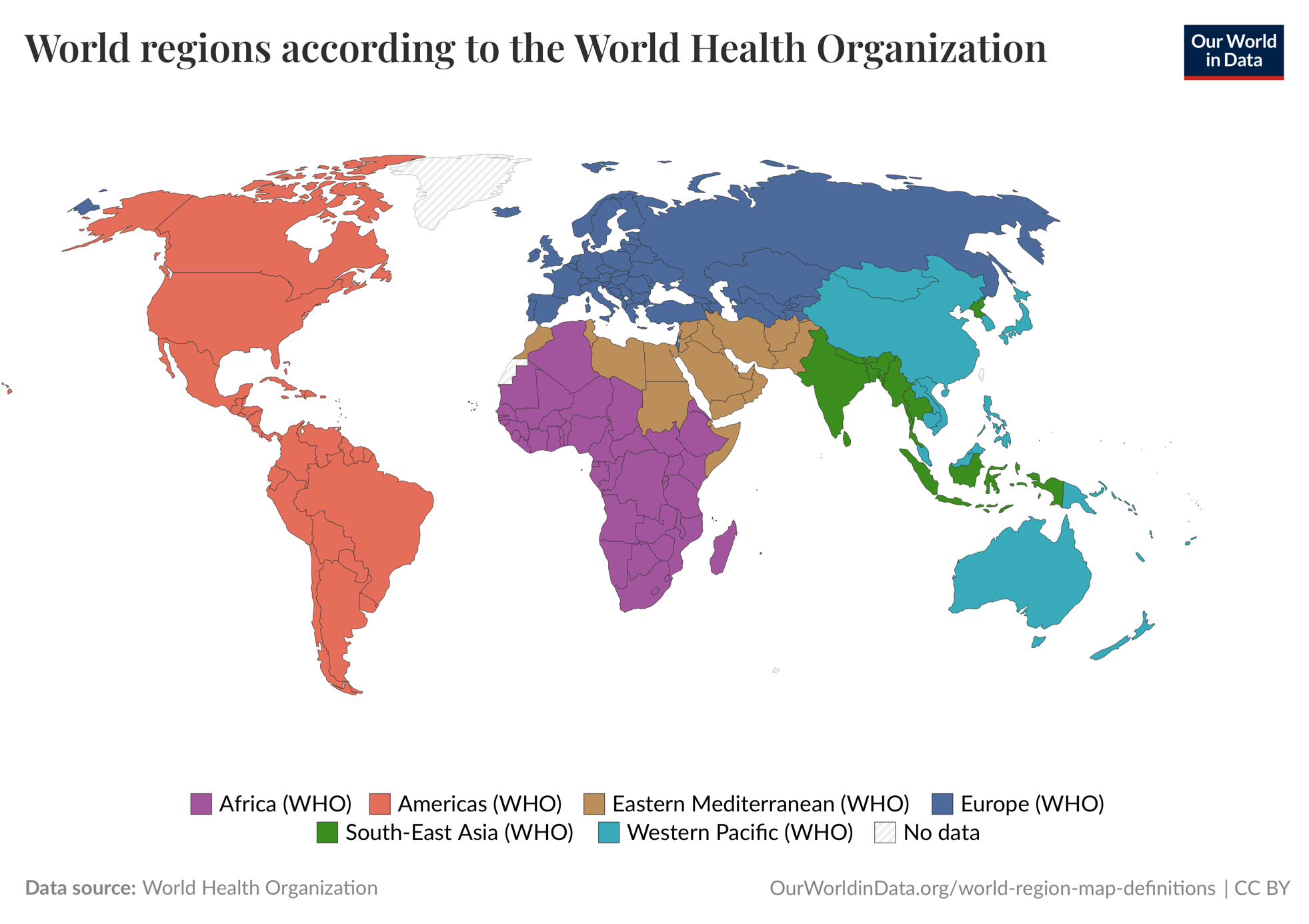


**Western Pacific
2.28%**

**Europe
5.90%**

**Americas
5.80%**

1. **Gestational Hypertension, Preeclampsia, and Eclampsia**
